# A universal fluorogenic switch for Fe(ii) ion based on N-oxide chemistry permits the visualization of intracellular redox equilibrium shift towards labile iron in hypoxic tumor cells†
†Electronic supplementary information (ESI) available. See DOI: 10.1039/c6sc05457a



**DOI:** 10.1039/c6sc05457a

**Published:** 2017-04-24

**Authors:** Tasuku Hirayama, Hitomi Tsuboi, Masato Niwa, Ayaji Miki, Satoki Kadota, Yukie Ikeshita, Kensuke Okuda, Hideko Nagasawa

**Affiliations:** a Laboratory of Pharmaceutical and Medicinal Chemistry , Gifu Pharmaceutical University , 1-25-4, Daigaku-nishi, Gifu-shi , Gifu , 501-1196 , Japan . Email: hirayamat@gifu-pu.ac.jp ; Email: hnagasawa@gifu-pu.ac.jp

## Abstract

Iron (Fe) species play a number of biologically and pathologically important roles. In particular, iron is a key element in oxygen sensing in living tissue where its metabolism is intimately linked with oxygen metabolism. Regulation of redox balance of labile iron species to prevent the generation of iron-catalyzed reactive oxygen species (ROS) is critical to survival. However, studies on the redox homeostasis of iron species are challenging because of a lack of a redox-state-specific detection method for iron, in particular, labile Fe^2+^. In this study, a universal fluorogenic switching system is established, which is responsive to Fe^2+^ ion based on a unique N-oxide chemistry in which dialkylarylamine N-oxide is selectively deoxygenized by Fe^2+^ to generate various fluorescent probes of Fe^2+^–CoNox-1 (blue), FluNox-1 (green), and SiRhoNox-1 (red). All the probes exhibited fluorescence enhancement against Fe^2+^ with high selectivity both in cuvette and in living cells. Among the probes, SiRhoNox-1 showed an excellent fluorescence response with respect to both reaction rate and off/on signal contrast. Imaging studies were performed showing the intracellular redox equilibrium shift towards labile iron in response to reduced oxygen tension in living cells and 3D tumor spheroids using SiRhoNox-1, and it was found that the hypoxia induction of labile Fe^2+^ is independent of iron uptake, hypoxia-induced signaling, and hypoxia-activated enzymes. The present studies demonstrate the feasibility of developing sensitive and specific fluorescent probes for Fe^2+^ with refined photophysical characteristics that enable their broad application in the study of iron in various physiological and pathological conditions.

## Abstract 2

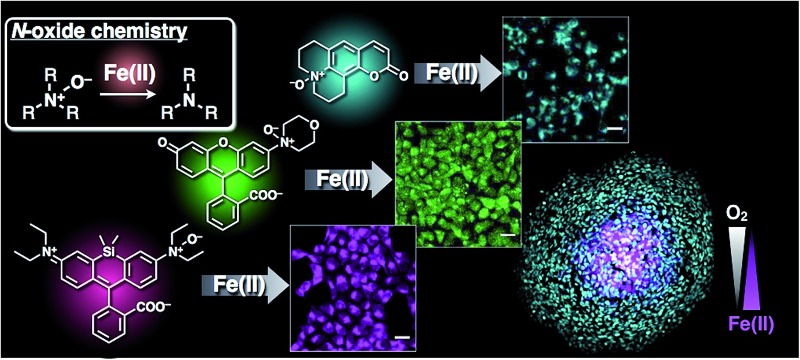
Oxygen-dependent fluctuation of labile Fe(ii) was visualized by a new N-oxide-based fluorescent probe for Fe(ii) ion.

## Introduction

Iron, the most abundant transition metal in our bodies, is involved in a number of biologically important processes such as respiration, oxygen transport, and energy production in collaboration with oxygen, based on its electrochemical properties with interconvertible multiple oxidation states.^1,2^ In living cells, iron exists mainly as ferrous (Fe^2+^) and ferric (Fe^3+^) ionic forms, the redox homeostasis of which is strictly regulated by iron-regulatory transcription factors and their related proteins.^3,4^ On the other hand, the chemical equilibrium between Fe^2+^/Fe^3+^ is susceptible to oxygen concentration in a neutral aqueous buffer and Fe^2+^ ion exists stably under anaerobic conditions especially in the presence of reductants.^5–8^ Intracellular labile iron, which is defined as iron species bound to small molecules or weakly bound to proteins, mainly consists of Fe^2+^ ion because of a high cellular abundance of reductants such as glutathione,^8,9^ existence of chaperones and transporters recognizing Fe^2+^,^10–12^ and high aqueous solubility of Fe^2+^.^13,14^ As a major contributor to oxidative damage of cells, Fe^2+^ is implicated in serious diseases such as cancers and neurodegenerative disorders, because of its ability to produce harmful reactive oxygen species *via* contact with oxygen, superoxide, and hydrogen peroxide (H_2_O_2_).^3,15^ Iron also has a key role as a sensor of cellular redox status, which is closely coupled with cellular oxygen sensing *via* regulation of expression and activation of various transcription factors and proteins sensitive to oxygen tension or redox states in cells such as hypoxia inducible factors (HIFs), heme oxygenases, and prolyl-hydroxylases among others.^16–18^ When cells suffer from low oxygen availability, various cellular signaling pathways are activated or inactivated to overcome the hypoxic stress. In particular, several tumor cells can adapt to the harsh conditions of hypoxia, resulting from immature angiogenesis in solid tumors, by developing sophisticated protective machinery against hypoxia through activation of HIF-1.^19–21^

To study the dynamics of intracellular iron in living cells, calcein^22,23^ and PhenGreen-SK^14,24,25^ have been widely used as fluorescent probes for Fe ions in living cells, and have facilitated development of the important concept of “labile iron species”. However, their turn-off response as well as poor metal selectivity limits their utility. The majority of other examples of fluorescent probes for Fe ions show response to Fe^3+^ and/or a turn-off readout,^26–29^ both of which characteristics are not suitable for measurement of the delicate alteration of labile iron consisting of Fe^2+^ in living cells. Recently, several redox state-selective fluorescent probes for Fe^2+^ with a turn-on readout have been developed.^30^ RhoNox-1, reported by our group, is the first example of detection of labile iron in live cells with a turn-on response, where N-oxide acts as a selective fluorogenic switch to Fe^2+^.^31^ Subsequently, Chang *et al.* reported IP-1 on the basis of a chelation-assisted oxidative C–O bond cleavage, which was successfully applied to detection of biologically stimulated accumulation of labile iron.^32^ Wang *et al.* reported that acetylhydroxylamine-modified naphthalimide, the N–O bond of which is cleaved by Fe^2+^ as in the case of N-oxide, could work as a fluorogenic probe for Fe^2+^ to visualize Zn-induced accumulation of Fe^2+^ as well as ischemia-mediated Fe^2+^ accumulation.^33^ Very recently, Wells and Renslo *et al.* reported a unique puromycin analogue, the activity of which is stimulated through O–O bond cleavage of trioxolane selectively by Fe^2+^. Using this analogue, the authors reported that cancer cells have higher labile Fe^2+^ level than non-cancerous cells.^34^ Among these successful strategies, our N-oxide chemistry-based approach offers the advantage of high selectivity for Fe^2+^. The previous examples of N-oxide based probes have a rhodamine scaffold,^31,35^ which limits their utility to only a single color region (*λ*_ex_ = 555 nm, *λ*_em_ = 575 nm).

Herein, development of a series of fluorescent probes of Fe^2+^ with emission at the blue (CoNox-1), green (FluNox-1 and FluNox-2), and deep-red (SiRhoNox-1) regions is reported, verifying the versatility of the N-oxide chemistry. The N-oxide chemistry-based approach, involving deoxygenation of dialkylarylamine N-oxide by Fe^2+^, worked well as a universal fluorogenic switch for all the dyes tested and the deep-red dye, SiRhoNox-1, showed a remarkable response among the probes. Moreover, application of SiRhoNox-1 to image the fluctuation of redox balance of Fe^2+^/Fe^3+^ in tumor cells under hypoxia revealed that redox equilibrium shifts towards Fe^2+^ along with a decrease in oxygen tension. This is the first example of use of a redox state-selective fluorescent probe for Fe^2+^ for elucidation of hypoxia-mediated alteration in the intracellular redox balance of labile iron.

## Results and discussion

### Design and synthesis of N-oxide-based fluorescent probes of Fe^2+^

**Scheme 1:**
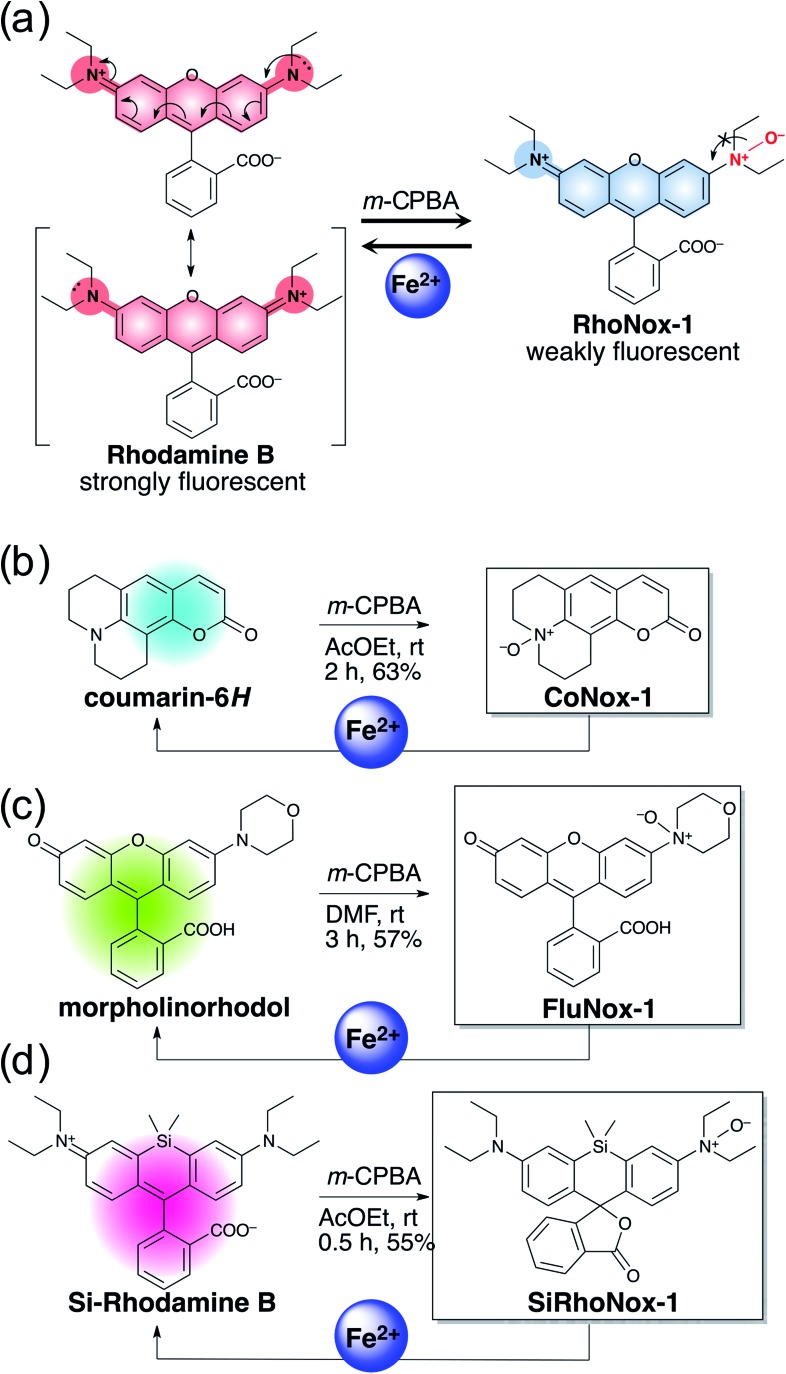
(a) Concept of fluorescence switching based on N-oxide chemistry represented by RhoNox-1. (b–d) Design and synthesis of various colored Fe^2+^ fluorescent probes based on N-oxide chemistry.

Previously, RhoNox-1 and HMRhoNox-M were reported to be useful fluorescent probes for detecting labile Fe^2+^ in living cells by exploiting N-oxide chemistry (Scheme 1a); however, their wavelength range is limited to the orange region (*λ*_ex_/*λ*_em_ = 555/575 nm).^31^ After careful consideration of the principle of fluorescence switching enabled by the N-oxide chemistry, it was found that isolation of a nitrogen atom from the π-conjugation system of xanthene chromophore by N–O bond formation at one of the dialkylarylamines is the critical factor for achieving the dark turn-off state of the probes (Scheme 1a) and an efficient turn-on response to Fe^2+^ during Fe^2+^-mediated deoxygenation. Using this principle, it was predicted that the N-oxide-based fluorogenic switch could be applicable to other fluorophore scaffolds bearing dialkylarylamine(s) in their π-conjugation system. To prove this concept, coumarin-6*H* (*λ*_ex_/*λ*_em_ = 403/495 nm), morpholinorhodol (510/535 nm), and Si-rhodamine B (645/660 nm) were chosen as parent fluorophores because they satisfied the following structural and photophysical requirements: (1) more than one dialkylarylamine(s) involved in their π-conjugation system, (2) high quantum yields under aqueous conditions, and (3) excitation/emission wavelength at blue, green, and deep-red regions. CoNox-1, FluNox-1, and SiRhoNox-1 were synthesized from coumarin-6*H*, morpholinorhodol, and Si-rhodamine B in modest yields, 63%, 57%, and 55%, respectively, by treating the corresponding dyes with *m*-CPBA. Diethylamino rhodol N-oxide was also synthesized as a rhodol-based probe, but its fluorescence response was not sufficient because of bright background signal as well as a low response rate (see ESI† on FluNox-2, *vide infra*). These results indicate that the N-oxidation reaction works universally for oxygenation of dialkylarylamines within fluorophores.

### Photophysical properties and fluorescence response of the probes

Photophysical properties of the probes as well as their parent fluorophores were evaluated (Table 1), and their performances were measured in physiological aqueous buffer system (50 mM HEPES buffer, pH 7.4). CoNox-1 exhibited an absorption peak at 295 nm (*ε* = 29 500 M^–1^ cm^–1^) with a shoulder at 335 nm, while coumarin-6*H* had an intense absorption at 402 nm with *ε* = 43 500 M^–1^ cm^–1^ (Fig. S1a†). Similarly, FluNox-1 and SiRhoNox-1 showed absorption peaks at 450 nm and 575 nm, respectively, with relatively or extremely small molar absorptions (*ε*) of 15 000 M^–1^ cm^–1^ and 1250 M^–1^ cm^–1^, while the corresponding parent dyes, morpholinorhodol and Si-rhodamine B have strong absorption bands at 510 nm with 48 500 M^–1^ cm^–1^ and 645 nm with 12 500 M^–1^ cm^–1^, respectively (Fig. S1b and c†). The hypsochromic shifts concomitant with a decrease in molar absorptions compared with the parent chromophores indicate that the N-oxidation resulted in isolation of the nitrogen atoms from π-conjugation of the fluorophores. Similar hypsochromic shifts were observed previously in the cases of RhoNox-1 and rhodamine B.^31^

**Table 1:** Photophysical properties of probes and their parent fluorophores in 50 mM HEPES buffer

Dye	*λ*_abs_ (nm)	*λ*_em_ (nm)	*ε* (M^–1^ cm^–1^)	*Φ* ^*a*^
Coumarin-6*H*	402	495	43 500	0.92
CoNox-1	295	495	29 500	0.04
Morpholinorhodol	510	535	48 500	0.15
FluNox-1	450	530	15 000	0.08
Si-rhodamine B	645	660	12 500	0.34
SiRhoNox-1	575	660	1250	0.08

^*a*^Quantum yields were determined in 50 nM HEPES buffer (pH 7.4).

**Fig. 1:**
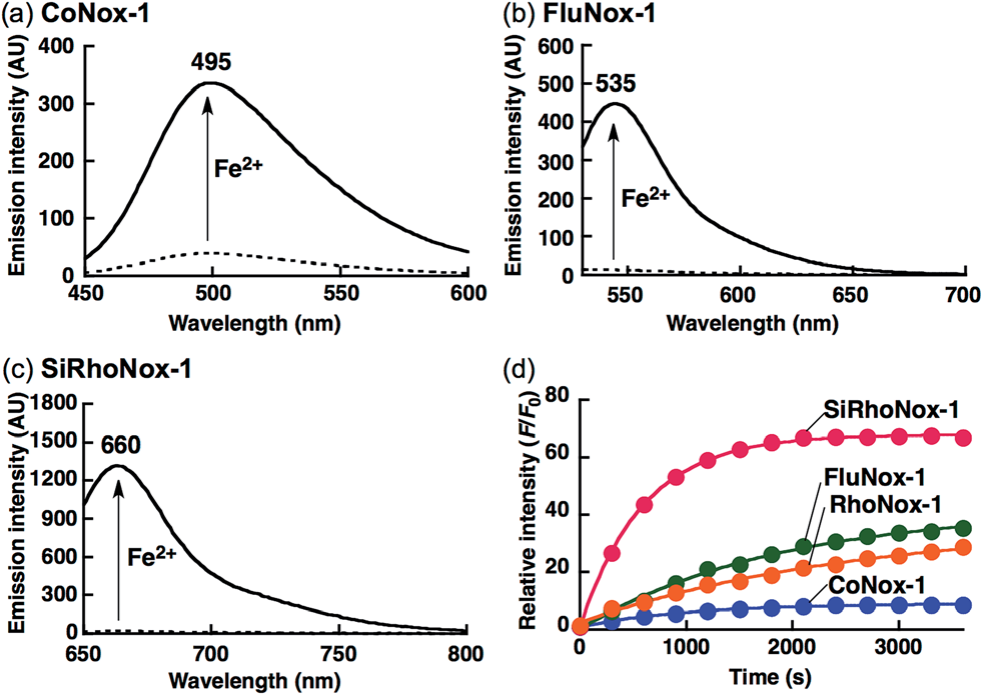
Fluorescence spectral change of (a) CoNox-1, (b) FluNox-1, and (c) SiRhoNox-1 on addition of FeSO_4_. Dashed and solid lines indicate spectra at 0 min (dashed lines) and 60 min (solid lines) after reaction with FeSO_4_, respectively. (d) Kinetics of fluorescence response of CoNox-1 (blue), FluNox-1 (green), RhoNox-1 (orange), and Si-RhoNox-1 (red). Relative fluorescence intensities are plotted every 300 s. All the data were acquired using a 2 μM probe concentration and 20 μM FeSO_4_ in 50 mM HEPES buffer (pH 7.4). *λ*_ex_ = (a) 405 nm, (b) 488 nm, and (c) 630 nm.

The N-oxide compounds showed considerably lower quantum yields (*Φ*) of 0.04 (CoNox-1), 0.08 (FluNox-1), and 0.08 (SiRhoNox-1), than the parent fluorophores; 0.92 (coumarin-6*H*), 0.15 (morpholinorhodol), and 0.34 (Si-rhodamine B) (Table 1). As suggested by their relatively low extinction coefficients and quantum yields, N-oxidation resulted in suppression of brightness (*Φ* × *ε*) as shown in their fluorescence spectra (dotted lines in Fig. 1a–c). Addition of Fe^2+^ to an aqueous solution of each of the probes triggered fluorescence spectral changes, with 10-, 30-, and 60-fold increases in maximum intensity for CoNox-1, FluNox-1, and SiRhoNox-1, respectively, suggesting that all the probes could detect Fe^2+^ with a turn-on response. It was also found that FluNox-2, derived from diethylaminorhodol as its parent fluorophore, did not show an efficient response against Fe^2+^ because of its slow reaction rate as well as high basal fluorescence (Fig. S2†). Among the probes, SiRhoNox-1 showed a distinctly fast response as well as a high off/on contrast. The response rates of the probes bearing diethylarylamine N-oxide structure were in the order of SiRhoNox-1 (*k*_obs_ = 1.7 × 10^–3^ s^–1^) > RhoNox-1 (*k*_obs_ = 7.0 × 10^–4^ s^–1^) > FluNox-2 (*k*_obs_ = n.d.). It is hypothesized that the fast response and high off/on fluorescence contrast of SiRhoNox-1 is attributed to its closed spirolactone form at neutral pH. In accordance with its extremely low extinction coefficient (1250 M^–1^ cm^–1^ at 575 nm) at neutral pH (Fig. S3c and f†), SiRhoNox-1 exists predominantly in spirolactone form (Scheme 1d), which shows significantly weak absorption in visible region. The spirolactonization causes no emission and thus has a low background signal because of a loss of π-conjugation, resulting in effective off/on contrast. Meanwhile, FluNox-2 exhibits strong absorbance (16 500 M^–1^ cm^–1^ at 450 nm, Fig. S3b and e†) resulting from its xanthene structure (see ESI†). Furthermore, it was found that N-oxide-based probes with a closed configuration at neutral pH tend to react with Fe^2+^ faster than those having an open quinoid configuration. For example, HMRhoNox-E,^35^ which is a hydroxymethylrhodamine derivative of RhoNox-1 and exists predominantly in spirocyclic form, showed a better reaction rate (*k*_obs_ = 7.0 × 10^–4^ s^–1^) than RhoNox-1, which exists predominantly in quinoid form (*k*_obs_ = 3.6 × 10^–4^ s^–1^) in neutral buffer. These factors synergistically improved the reaction kinetics and off/on contrast of SiRhoNox-1 for Fe^2+^.

Based on the measured absorbance spectral change and extinction coefficients of each of the probes and their parent dyes, CoNox-1, FluNox-1, and SiRhoNox-1 were converted to the corresponding fluorescent compounds at approximately 35%, 17%, and 90%, respectively, after incubation with Fe^2+^ for 1 h (Fig. S4†). LC-MS analysis of the reaction mixtures of each of the probes and Fe^2+^ revealed that the de-oxygenation reaction proceeded almost exclusively to produce the corresponding dyes (Fig. S5†). A trace amount of byproducts in which the ethyl group was cleaved, was observed in the case of SiRhoNox-1. The generation of these byproducts might be attributed to Fe-induced oxidative dealkylation.^36,37^

**Fig. 2:**
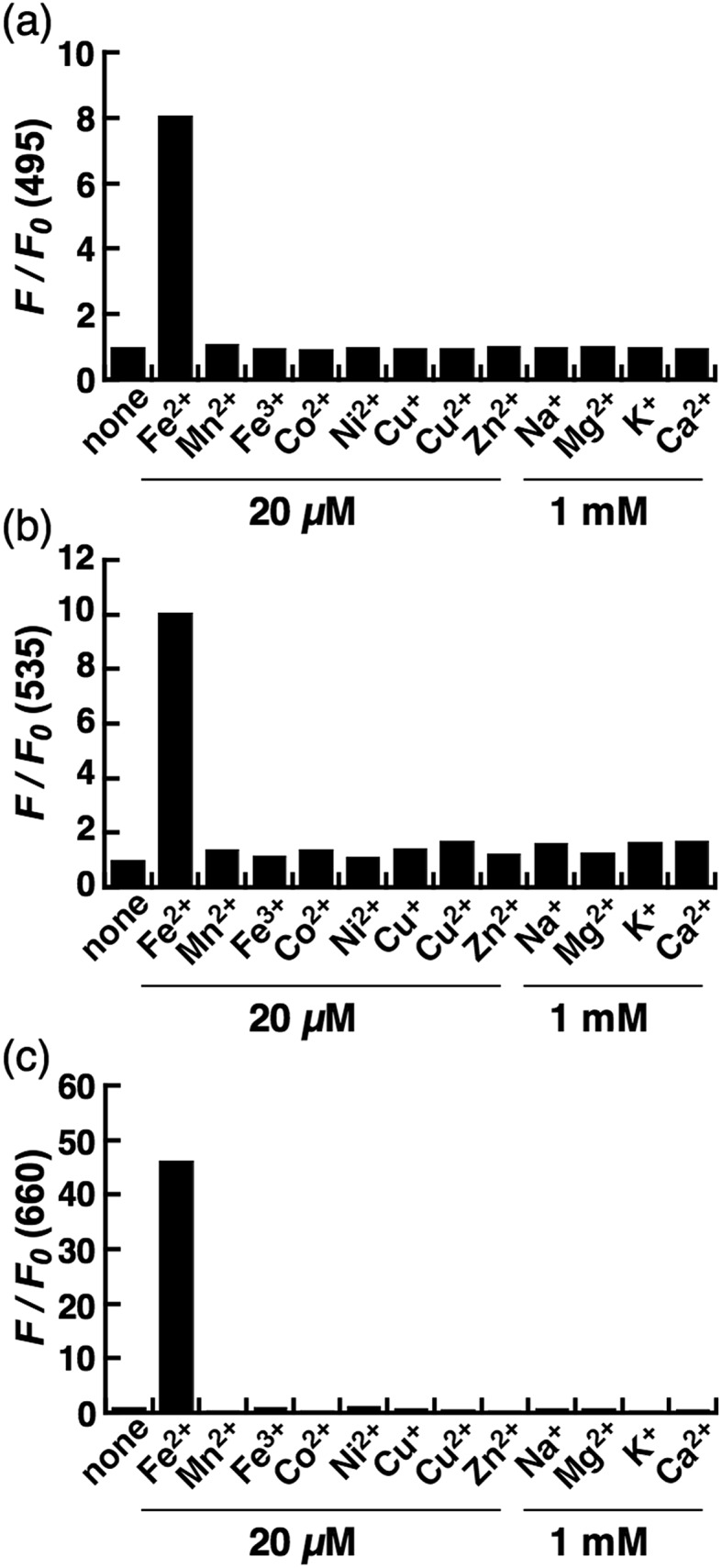
Fluorescence response of 2 μM (a) CoNox-1, (b) FluNox-1, and (c) SiRhoNox-1 against various metal ions (1 mM of alkali and alkaline earth metal ions, and 20 μM of all other metal ions). All the data were acquired 1 h after reaction with the indicated metal ions in 50 mM HEPES buffer (pH 7.4, 0.2% DMSO). Bars represent relative fluorescence intensities at 495 nm (a), 535 nm (b), and 660 nm (c); *λ*_ex_ = 405 nm (a), 488 nm (b), and 630 nm (c).

The biggest advantage of an N-oxide-based strategy is the high metal selectivity for Fe^2+^. Fig. 2 depicts metal selectivity data for the probes against biologically relevant first row transition metal ion species, alkali, and alkaline earth metal ion species. All three probes (CoNox-1, FluNox-1, and SiRhoNox-1) showed prominent fluorescence response to Fe^2+^ only, while other metal ions including Fe^3+^, alkali, and alkaline earth metal ions did not trigger any increase in fluorescence. Moreover, none of the probes exhibited a significant fluorescence response against biologically relevant reductants such as glutathione and ascorbate as well as reactive oxygen species (Fig. S6†), suggesting that all three probes have high selectivity for Fe^2+^ and adequate stability for biological applications. The parent fluorophores also showed good stability against highly reactive oxygen species such as hydroxyl radical and hypochlorite, which potentially oxidize alkylarylamines (Fig. S7†). Overall, it was established that the design strategy based on N-oxide chemistry works as a universal fluorogenic molecular switching system selective for Fe^2+^ using various fluorophores bearing a dialkylarylamine in a π-conjugation system.

### Live-cell imaging experiments

**Fig. 3:**
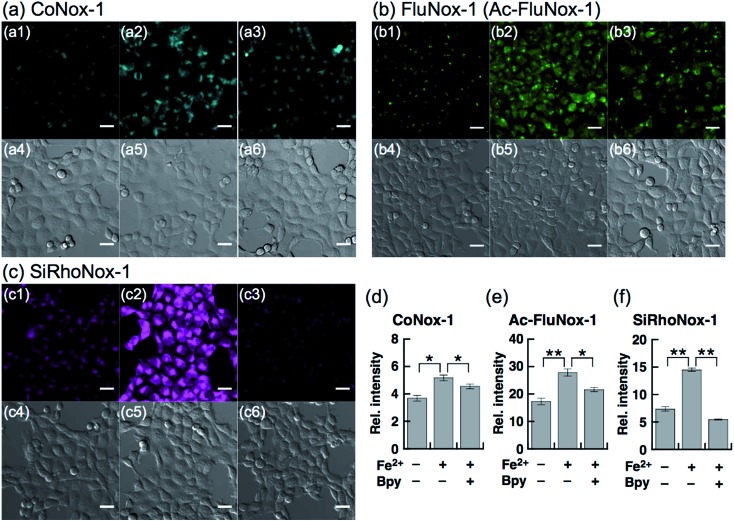
Confocal fluorescence microscopy analysis for detection of Fe^2+^ in HepG2 cells using (a) CoNox-1, (b) Ac-FluNox-1, and (c) SiRhoNox-1. (1) Images of the cells treated with probe for 2 h (CoNox-1) or 1 h (Ac-FluNox-1 and SiRhoNox-1). (2) Images of the cells supplemented with Fe^2+^ for 30 min prior to probe. (3) Images of the cells treated with Fe^2+^ prior to probe in the presence of 2,2′-bipyridyl (Bpy). (4–6) Differential interference contrast (DIC) images for the same microscopic fields as (1–3). (d) Quantification of data in (a1–a3). (e) Quantification of data in (b1–b3). (f) Quantification of data in (c1–c3). Statistical analyses were performed with a Student's *t*-test. **P < 0.01, *P < 0.05 (*n* = 3). Error bars indicate ± S.E.M. All the data were acquired with 5 μM probe, 1 mM Bpy, and 100 μM Fe^2+^ [supplemented as (NH_4_)_2_Fe(SO_4_)_2_·6H_2_O (FAS)]. Scale bars indicate 25 μm.

Next, a live-cell imaging study was performed with the probes. Although treatment with CoNox-1 for 1 h was not enough to gain detectable signal because of its high hydrophilicity and low off/on contrast, prolonging the treatment time up to 2 h caused significant signal enhancement in Fe^2+^-treated cells compared with the control cells (Fig. 3a1 and a2). Meanwhile, 1 h treatment was sufficient to obtain adequate signals for the other two probes. FluNox-1 was impermeable across the cellular membrane, and thus it was converted to a cell-permeable analogue, Ac-FluNox-1, by acetylation of the hydroxyl group of morpholinorhodol followed by N-oxidation (see ESI†). CoNox-1, Ac-FluNox-1, and SiRhoNox-1 showed significantly higher signals in Fe^2+^-treated cells than in the control cells (Fig. 3a1/a2, b1/b2 and c1/c2). In particular, distinctly high signal contrast was observed between the control and Fe^2+^-treated cells in the cells stained by SiRhoNox-1 (Fig. 3c1/c2). The Fe^2+^-induced enhancements of fluorescence signal were completely suppressed to the basal level by treatment with Bpy (2,2′-bipyridyl), which acts as a Fe^2+^ ion chelator (Fig. 3a2/a3, b2/b3 and c2/c3).^22,25^ Although CoNox-1 and Ac-FluNox-1 were not sensitive enough to detect endogenous labile Fe^2+^ (Fig. S8a and b†), the cells treated with SiRhoNox-1 in the presence of Bpy without supplementation of Fe^2+^ exhibited significantly lower signal than the basal level (Fig. S8c†), suggesting that SiRhoNox-1 could detect endogenous labile Fe^2+^. These results are consistent with the observation of distinctly high sensitivity of Si-RhoNox-1 compared with the other dyes in the cuvette (Fig. 1d). Ac-FluNox-1 showed cytosolic localization with punctate staining pattern of cells (Fig. S9b†), while CoNox-1 and SiRhoNox-1 have a similar localization pattern, which was identical with endoplasmic reticulum (ER)-staining dyes (Fig. S9a and c†).

### Detection of intracellular Fe^2+^ by flow cytometry

**Fig. 4:**
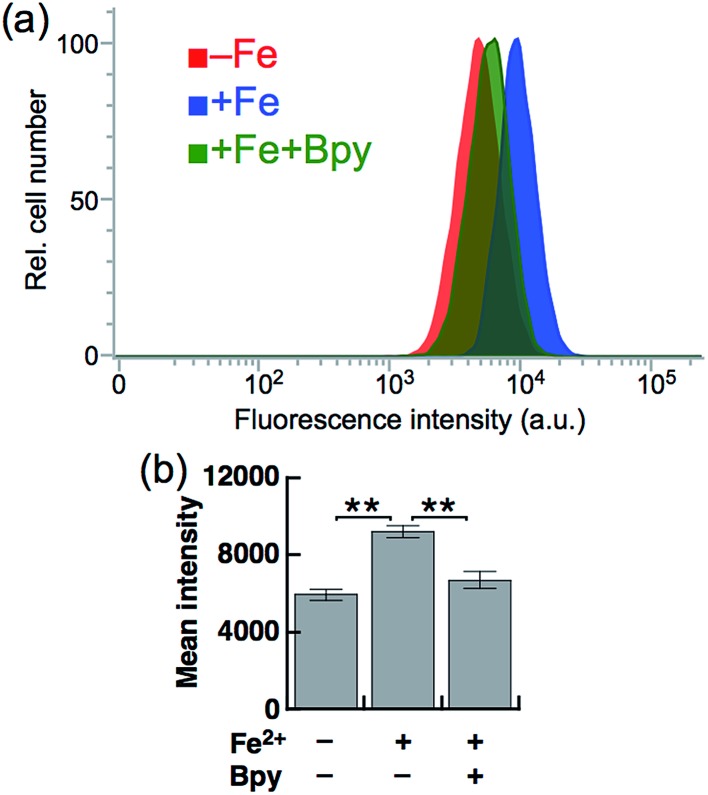
(a) Representative flow cytometry histograms of control cells treated with 5 μM SiRhoNox-1 for 1 h (red), cells treated with 100 μM Fe^2+^ (FAS) for 30 min prior to staining with 5 μM SiRhoNox-1 for 1 h (blue), and cells treated with 100 μM Fe^2+^ (FAS) for 30 min prior to staining with 5 μM SiRhoNox-1 in the presence of 1 mM Bpy for 1 h (green). A total of 10 000 cells was analyzed for each histogram. (b) Mean intensities of fluorescence observed by flow cytometry. Statistical analyses were performed with a Student's *t*-test. **P < 0.01 (*n* = 5). Error bars indicate ± S.E.M.

Next, SiRhoNox-1 was applied to flow cytometry analysis of subcellular labile Fe^2+^. As observed in the imaging study, when the cells were supplemented with Fe^2+^ the population distribution shifted from a low fluorescence signal region to a high signal region [Fig. 4a (red/blue) and Fig. 4b]; the shift was negated by treatment with Bpy [Fig. 4a (blue/green) and Fig. 4b]. However, in contrast to the imaging study, flow cytometry analysis was not sensitive enough to detect endogenous labile Fe^2+^. It is posited that instrumentation limit or a sample preparation factor, such as treatment with trypsin, resulted in the relatively low sensitivity of flow cytometry analysis compared with microscopic analysis of live cells.

### Detection of hypoxia-induced fluctuation of labile Fe^2+^

**Fig. 5:**
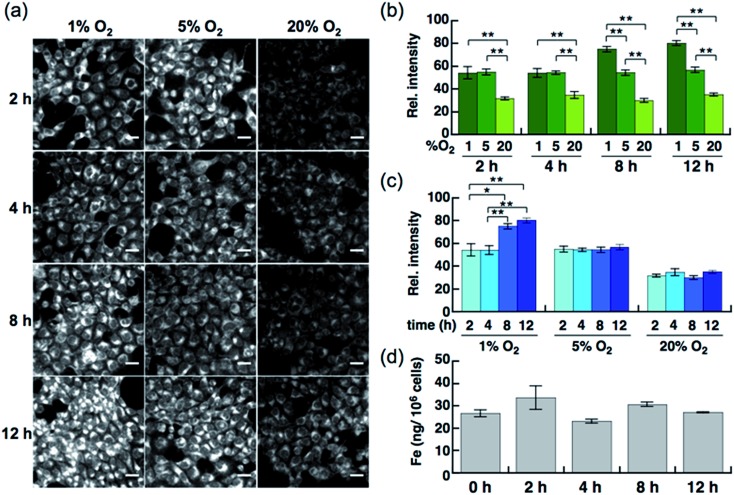
(a) Confocal fluorescence microscopy images of HepG2 cells after incubation for 2 h, 4 h, 8 h, and 12 h under various oxygen concentrations (1%, 5%, and 20%). All the cells were stained with 5 μM SiRhoNox-1. Scale bars indicate 25 μm. (b) Quantification and evaluation of fluorescence signals at each oxygen concentration (1% O_2_, 5% O_2_, and 20% O_2_) at each time point. (c) Quantification and evaluation of time-dependency (2, 4, 8, and 12 h) at each oxygen concentration. Statistical analyses were performed with a Student's *t*-test. **P < 0.01, *P < 0.05 (*n* = 4). Error bars indicate ± S.E.M. (d) Measurement of total iron contents of the cells (per 10^6^ cells) incubated under 1% O_2_ by atomic absorption. No significant increase was observed (*n* = 3).

Live-cell imaging experiments and the flow cytometry analysis revealed SiRhoNox-1 to be the most efficient and promising fluorescent sensor for Fe^2+^. Thus, SiRhoNox-1 was applied to monitor minute fluctuations of labile Fe^2+^ during cellular stress response. Previously, it was reported that oxidative stress causes fluctuation of redox balance of Fe^2+^ during light-induced retinal cell death in a cellular model of aged-macular degradation.^38^ As stability of Fe^2+^ in aqueous buffer is highly dependent on concentration of dissolved oxygen,^6,8^ it was hypothesized that intracellular redox balance between Fe^2+^/Fe^3+^ could be altered under hypoxic conditions. An imaging study was conducted to evaluate intracellular fluctuation of labile Fe^2+^ under various oxygen concentrations (1%, 5%, and 20% O_2_) at various time points (2 h, 4 h, 8 h, and 12 h) using SiRhoNox-1 as an indicator of labile Fe^2+^ (Fig. 5). As expected, distinctly higher fluorescence signals were observed after incubation for just 2 h under 1% and 5% O_2_ compared with 20% O_2_ (Fig. 5a and b). The fluorescence signals were enhanced under hypoxia (1% and 5% O_2_) at all time points (Fig. 5a and c). The signal enhancement plateaued at 8 h when the cells were incubated under 1% O_2_, while similar signal enhancements were observed at each time point under 5% O_2_.

To evaluate whether the signal increase was caused by an alteration of the redox balance of the labile Fe^2+^ level, Bpy was employed as a Fe^2+^ chelator and diphenyliodonium chloride (DPI) as an inhibitor of NADPH-dependent reductases^39–41^ such as NADPH-cytochrome c reductase, the enzymatic activity of which is strongly activated under hypoxic conditions. In the presence of Bpy, the signal enhancement was completely suppressed for cells incubated under 1% and 5% O_2_ (Fig. S10a–c†), indicating that the observed enhancements in signal were distinctly triggered by labile Fe^2+^ under hypoxic conditions. Prior to employing DPI as an inhibitor of reductases in cells, it was confirmed that SiRhoNox-1 is inert against nitroreductase even in the presence of excess NADPH in cuvette (Fig. S11†). As observed in the cuvette assays, DPI did not interfere with the hypoxia-triggered fluorescence response in cells, suggesting that intracellular NADPH-dependent reductases including nitroreductase did not contribute to enhancement of the fluorescence signal (Fig. S12†). This behavior is distinctly different from the previously reported hypoxia-sensitive fluorescent probes, the detection mechanisms for which are based on hypoxia-specific enzymatic reduction.^42–48^ Contrary to previous reports that hypoxia regulates the ferrous iron uptake *via* up-regulation of divalent metal ion transporter 1 (DMT1),^49–51^ a direct importer of Fe^2+^ ion, no significant increase was observed in total iron contents of the cells incubated under hypoxia (1% O_2_), as quantified by atomic absorption spectroscopy (Fig. 5d). These findings clearly demonstrate that the observed up-regulation of labile Fe^2+^ levels induced by hypoxia is independent of total cellular iron uptake but dependent on shift of Fe^2+^/Fe^3+^ chemical equilibria in cells. Moreover, the change of redox balance may not be mediated by HIF-mediated protein expression at an early stage (2 h). Western blot analysis of HIF-1α showed that the protein expression level of HIF-1α increased and reached a maximum at 4 h under 1% O_2_; no significant protein expression of HIF-1α was observed in the cells incubated under 5% O_2_ (Fig. S13†) while a stabilization of Fe^2+^ was observed by the fluorescence imaging at any time point tested for cells incubated under 5% O_2_ (Fig. 5a and c). The stabilization of Fe^2+^ under low oxygen concentrations as generally observed in cuvette may also occur in living cells. These results suggests that the intracellular redox balance between Fe^2+^/Fe^3+^ occurs independently of a well-known cellular response to hypoxia such as HIF-regulated signaling.^21^ Accordingly, visualization was successful of the intracellular redox equilibrium shift towards labile Fe^2+^ in response to reduced oxygen tensions in tumor cells using a novel Fe^2+^ selective fluorescent probe, SiRhoNox-1. The hypoxia-induced redox shift of labile iron was also observed in 3D cultured HepG2 spheroids (Fig. S14†). The spheroids with diameters of approximately 500 μm cultured for 5 days contained regions of hypoxia detected by immunochemical analysis using the hypoxic marker pimonidazole (PIMO).^52,53^ The fluorescent images of a central slice of the spheroids treated by SiRhoNox-1 showed specific staining around a central hypoxic core. Taken together, the new Fe^2+^-selective fluorescent probe, SiRhoNox-1 provided an insight into hypoxia-induced up-regulation of labile Fe^2+^ level in a time- and O_2_-concentration-dependent manner and revealed that the elevation of labile Fe^2+^ levels is caused by an alteration in redox balance of the Fe ion in hypoxic cells. Although the imaging study of the spheroids required a sequence of fixation process, embedding, and sectioning process, the fluorescence signal from SiRhoNox-1 was retained with a detectable level, indicating that SiRhoNox-1 could be applicable not only to living cells but also to fixed and sectioned samples. The source of labile iron is likely to be intracellular ferritin, which stores thousands of iron ions in one protein and releases labile iron^54–56^ by responding to acute cellular requirements of iron independent of any protein expression. Actually, it was observed that hypoxic treatment (1%O_2_) did not affect intracelluar ferritin level (Fig. S15†), supporting the idea that up-regulation of Fe^2+^ level mainly depends on alteration of intracellular equilibrative system for iron homeostasis but not on ferritin degradation. Further investigation is necessary to explore the regulation of iron metabolism depending on the intracellular redox environment in cancer cells.

## Conclusion

Using N-oxide chemistry a color series of Fe^2+^-fluorescent probes was established. N-oxidation of dialkylarylamine involved in the π-conjugation system of the chromophores could convert each chromophore to a Fe^2+^-selective fluorescent probe, and the principle worked for a wide range of fluorophores to generate CoNox-1 (coumarin), FluNox-1 (rhodol), and SiRhoNox-1 (Si-rhodamine B) in addition to the previously described probe, RhoNox-1 (Rhodamine B). All the probes presented here exhibited a selective turn-on response against Fe^2+^ in aqueous buffer, with SiRhoNox-1 characterized by a distinctly high off/on contrast as well as preferred reaction kinetics. All the probes were applicable to live-cell imaging, and SiRhoNox-1 also showed extremely high signal/background ratio and good response rate within cells. Using SiRhoNox-1, it was observed that the redox balance of labile Fe species is altered under hypoxic conditions and that the up-regulation of labile Fe^2+^ is clearly dependent on O_2_ level but independent of total cellular amount of iron (iron uptake), ferritin degradation, HIF-1α-mediated signal transduction, and hypoxia-activated enzymes. Furthermore, it was demonstrated that these phenomena occurred in the central hypoxic core of the 3D spheroid tumor models by means of SiRhoNox-1. This is the first fluorescent imaging tool capable of capturing a slight equilibrium shift of cellular redox balance to labile Fe^2+^ under hypoxia. Successful development of this series of Fe^2+^-selective fluorescent probes with various color emissions on the basis of the N-oxide chemistry would encourage and enable the progress of biological study in iron-related physiological and pathological events.

## References

[cit1] Crichton R. R., Wilmet S., Legssyer R., Ward R. J. (2002). J. Inorg. Biochem..

[cit2] Lippard S. J. and Berg J. M., Principles of Bioinorganic Chemistry, University Science Books, 1997.

[cit3] Hentze M. W., Muckenthaler M. U., Galy B., Camaschella C. (2010). Cell.

[cit4] Wang J., Pantopoulos K. (2011). Biochem. J..

[cit5] Stumm W., Lee G. F. (1961). Ind. Eng. Chem..

[cit6] Sung W., Morgan J. J. (1980). Environ. Sci. Technol..

[cit7] Krishnamurti G. S. R., Huang P. M. (1991). Clays Clay Miner..

[cit8] Hider R. C., Kong X. L. (2011). BioMetals.

[cit9] Hider R. C., Kong X. (2013). Dalton Trans..

[cit10] Bluteau A.-L., O'Neill H. A., Kennedy M. C., Ikeda-Saito M., Isaya G., Szweda L. I. (2004). Science.

[cit11] Shi H., Bencze K. Z., Stemmler T. L., Philpott C. C. (2008). Science.

[cit12] Yanatori I., Yasui Y., Tabuchi M., Kishi F. (2014). Biochem. J..

[cit13] Cabantchik Z. I. (2014). Front. Pharmacol..

[cit14] Kakhlon O., Cabantchik Z. I. (2002). Free Radic. Biol. Med..

[cit15] Halliwell B., Gutteridgeb J. M. C. (1992). FEBS Lett..

[cit16] Simpson R. J., McKie A. T. (2015). Metallomics.

[cit17] Acker T., Fandrey J., Acker H. (2006). Cardiovasc. Res..

[cit18] Haddad J. J. (2002). Respir. Res..

[cit19] Vaupel P. (2004). Semin. Radiat. Oncol..

[cit20] Dewhirst M. W., Cao Y., Moeller B. (2008). Nat. Rev. Cancer.

[cit21] Semenza G. L. (2012). Cell.

[cit22] Breuer W., Epsztejn S., Cabantchik Z. I. (1995). J. Biol. Chem..

[cit23] Thomas F., Serratrice G., Béguin C., Aman E. S., Pierre J. L., Fontecave M., Laulhère J. P. (1999). J. Biol. Chem..

[cit24] Petrat F., Weisheit D., Lensen M., De Groot H., Sustmann R., Rauen U. (2002). Biochem. J..

[cit25] Petrat F., de Groot H., Rauen U. (2000). Arch. Biochem. Biophys..

[cit26] Carter K. P., Young A. M., Palmer A. E. (2014). Chem. Rev..

[cit27] Que E. L., Domaille D. W., Chang C. J. (2008). Chem. Rev..

[cit28] Sahoo S. K., Sharma D., Bera R. K., Crisponi G., Callan J. F. (2012). Chem. Soc. Rev..

[cit29] Zhu H., Fan J., Wang B., Peng X. (2015). Chem. Soc. Rev..

[cit30] Hirayama T., Nagasawa H. (2017). J. Clin. Biochem. Nutr..

[cit31] Hirayama T., Okuda K., Nagasawa H. (2013). Chem. Sci..

[cit32] Au-Yeung H. Y., Chan J., Chantarojsiri T., Chang C. J. (2013). J. Am. Chem. Soc..

[cit33] Xuan W., Pan R., Wei Y., Cao Y., Li H., Liang F.-S., Liu K.-J., Wang W. (2016). Bioconjug. Chem..

[cit34] Spangler B., Morgan C. W., Fontaine S. D., Vander Wal M. N., Chang C. J., Wells J. A., Renslo A. R. (2016). Nat. Chem. Biol..

[cit35] Niwa M., Hirayama T., Okuda K., Nagasawa H. (2014). Org. Biomol. Chem..

[cit36] Nehru K., Seo M. S., Kim J., Nam W. (2007). Inorg. Chem..

[cit37] Kok G. B., Pye C. C., Singer R. D., Scammells P. J. (2010). J. Org. Chem..

[cit38] Imamura T., Hirayama T., Tsuruma K., Shimazawa M., Nagasawa H., Hara H. (2014). Exp. Eye Res..

[cit39] Tew D. G. (1993). Biochemistry.

[cit40] Chakraborty S., Massey V. (2002). J. Biol. Chem..

[cit41] Riganti C., Gazzano E., Polimeni M., Costamagna C., Bosia A., Ghigo D. (2004). J. Biol. Chem..

[cit42] Tanabe K., Hirata N., Harada H., Hiraoka M., Nishimoto S.-i. (2008). Chembiochem.

[cit43] Nakata E., Yukimachi Y., Kariyazono H., Im S., Abe C., Uto Y., Maezawa H., Hashimoto T., Okamoto Y., Hori H. (2009). Bioorg. Med. Chem..

[cit44] Kiyose K., Hanaoka K., Oushiki D., Nakamura T., Kajimura M., Suematsu M., Nishimatsu H., Yamane T., Terai T., Hirata Y., Nagano T. (2010). J. Am. Chem. Soc..

[cit45] Komatsu H., Harada H., Tanabe K., Hiraoka M., Nishimoto S.-i. (2010). MedChemComm.

[cit46] Okuda K., Okabe Y., Kadonosono T., Ueno T., Youssif B. G. M., Kizaka-Kondoh S., Nagasawa H. (2012). Bioconjug. Chem..

[cit47] Takahashi S., Piao W., Matsumura Y., Komatsu T., Ueno T., Terai T., Kamachi T., Kohno M., Nagano T., Hanaoka K. (2012). J. Am. Chem. Soc..

[cit48] Piao W., Tsuda S., Tanaka Y., Maeda S., Liu F., Takahashi S., Kushida Y., Komatsu T., Ueno T., Terai T., Nakazawa T., Uchiyama M., Morokuma K., Nagano T., Hanaoka K. (2013). Angew. Chem., Int. Ed. Engl..

[cit49] Li Z., Lai Z., Ya K., Fang D., Ho Y. W., Lei Y., Ming Q. Z. (2008). J. Cell. Mol. Med..

[cit50] Qian Z. M., Mei Wu X., Fan M., Yang L., Du F., Yung W. H., Ke Y. (2011). J. Cell. Physiol..

[cit51] Wang D., Wang L. H., Zhao Y., Lu Y. P., Zhu L. (2010). IUBMB life.

[cit52] Nozawa-Suzuki N., Nagasawa H., Ohnishi K., Morishige K. (2015). Biochem. Biophys. Res. Commun..

[cit53] Gomes A., Guillaume L., Grimes D. R., Fehrenbach J., Lobjois V., Ducommun B. (2016). PLoS One.

[cit54] Lai M. I., Feng L. W., Yap B. K., George E., Abdullah M. (2015). Int. J. Biosci. Biochem. Bioinforma..

[cit55] Melman G., Bou-Abdallah F., Vane E., Maura P., Arosio P., Melman A. (2013). Biochim. Biophys. Acta.

[cit56] Thomas C. E., Aust S. D. (1986). J. Biol. Chem..

