# Supplementary material for: A universal fluorogenic switch for Fe(ii) ion based on N-oxide chemistry permits the visualization of intracellular redox equilibrium shift towards labile iron in hypoxic tumor cells
Source: Chem Sci. 2017 Apr 24;8(7):4858–66. doi: 10.1039/c6sc05457a (PMC5603896; doi:10.1039/c6sc05457a)
Supplement: Supplementary file 1 [file SC-008-C6SC05457A-s001.pdf]

Electronic Supplementary Material (ESI) for Chemical Science.
This journal is © The Royal Society of Chemistry 2017

Supporting information

A universal fluorogenic switch for Fe(II) ion based on *N*-oxide chemistry permits visualization of intracellular redox equilibrium shift towards labile iron in hypoxic tumor cells

Tasuku Hirayama,[1] Hitomi Tsuboi,[1] Masato Niwa,[1] Satoki Kadota,[1] Ayaji Miki,[1] Kensuke Okuda,[1,2] and Hideko Nagasawa[1]

[1]Laboratory of Pharmaceutical and Medicinal Chemistry, Gifu Pharmaceutical University

[2]Present address: Laboratory of Bioorganic & Natural Products Chemistry, Kobe Pharmaceutical University

Contents

General S2
1. Synthesis of a series of *N*-oxide-based fluorescent probes S2
2. Steady-state absorption and fluorescence spectroscopy S6
3. Product analysis S7
4. Cell culture experiments S7
5. Confocal fluorescence imaging experiments S8
6. Imaging experiments under hypoxia S8
7. Quantification of total intracellular iron by atomic absorption spectrometry S9
8. Western blotting of HIF-1α and ferritin S9
9. Preparation and imaging study of spheroids S9
Figure S1 Absorption spectra of the probes and the parent dyes S11
Figure S2 Photophysical properties and response of FluNox-2 .... S11
Figure S3 Absorption spectral changes of the probes against pH S12
Figure S4 Absorption spectral changes of the probes against Fe^{2+} S12
Figure S5 Product analyses by HPLC-IT-TOF mass S13
Figure S6 Stability tests under reductants and oxidants S14
Figure S7 Stability tests of the parent fluorophores S14
Figure S8 Fluorescence imaging of HepG2 cells for detection of endogenous Fe^{2+} S15
Figure S9 Co-staining experiments S15
Figure S10 Fluorescence imaging under hypoxia in the presence of 2,2'-bipyridyl S16
Figure S11 Fluorescence response of SiRhoNox-1 against nitroreductase S16
Figure S12 Fluorescence response of SiRhoNox-1 in the presence of DPI S17
Figure S13 Western blot analysis of HIF-1α in HepG2 cells S17
Figure S14 Confocal fluorescence images of spheroids of HepG2 cells S17
Figure S15 Western blot analysis of ferritin in HepG2 cells S18
References S18
^{1}H- and ^{13}C-NMR spectra of the newly synthesized compounds S19

General: All chemicals used in this study were commercial products of the highest available purity and were further purified by the standard methods, if necessary. ^{1}H-NMR spectra were obtained on a JEOL ECA-500 spectrometer at 500 MHz and JEOL JNM AL-400 spectrometer at 400 MHz. ^{13}C-NMR spectra were obtained on a JEOL ECA-500 spectrometer at 125 MHz and JEOL AL-400 spectrometer at 100 MHz. Chemical shifts of ^{1}H-NMR are referenced to tetramethylsilane (TMS). Chemical shifts of ^{13}C-NMR are referenced to $CDCl_3$ (77.0) or CD_3OD (49.0). Chemical shifts and coupling constants were recorded in units of ppm and Hz, respectively. ESI-mass spectra were measured on a JEOL JMS-T100TD mass spectrometer. High-resolution mass spectra (HRMS) were measured on a JEOL JMS-T100TD by using polyethyleneglycol (PEG) as an internal standard. Reactions were monitored by silica gel TLC (Merck Silica gel 60 PF_{254}) with visualization of components by UV light (254 nm) or with visual observation of the dye spots. Products were purified on a silica gel column chromatography (Taiko-shoji AP-300S).

1. Synthesis of a series of *N*-oxide-based fluorescent probes.

CoNox-1

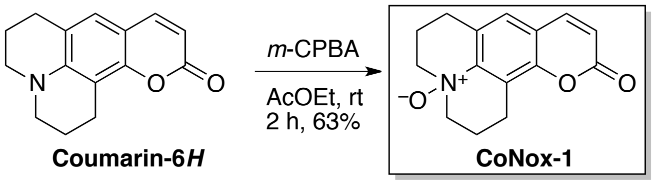

CoNox-1. Coumarin-6*H* was synthesized as previously described.[1] Coumarin-6*H* (56 mg, 0.23 mmol) was dissolved in ethyl acetate (10 mL), and then *m*-CPBA (44 mg, 0.26 mmol) was added at 0 °C. After stirring at room temperature for 2 h, the mixture was filtered by a pad of celite. The filtrate was evaporated, and the residue was purified by silica gel column chromatography ($CHCl_3$: MeOH = 6:1 to 4:1) to give CoNox-1 as pale yellow solid (37 mg, 63%).

^{1}H NMR (500 MHz, CD_3OD): δ 7.86 (d, J = 9.4 Hz, 1H), 7.38 (s, 1H), 6.45 (d, J = 9.4 Hz, 1H), 3.84–3.77 (m, 2H), 3.64–3.61 (m, 2H), 3.19–2.93 (m, 6H), 2.15–2.05 (m, 2H); ^{13}C-NMR (125 MHz, CD_3OD): δ 161.9, 151.2, 145.8, 144.7, 128.2, 127.4, 119.84, 119.81, 118.2, 67.1, 66.9, 25.7, 20.5, 17.2, 16.4; HRMS (ESI+) *m/z* calcd for $C_{15}H_{16}NO_3^{+}$: 258.1125, found: 258.1136.

FluNox-1 and Ac-FluNox-1

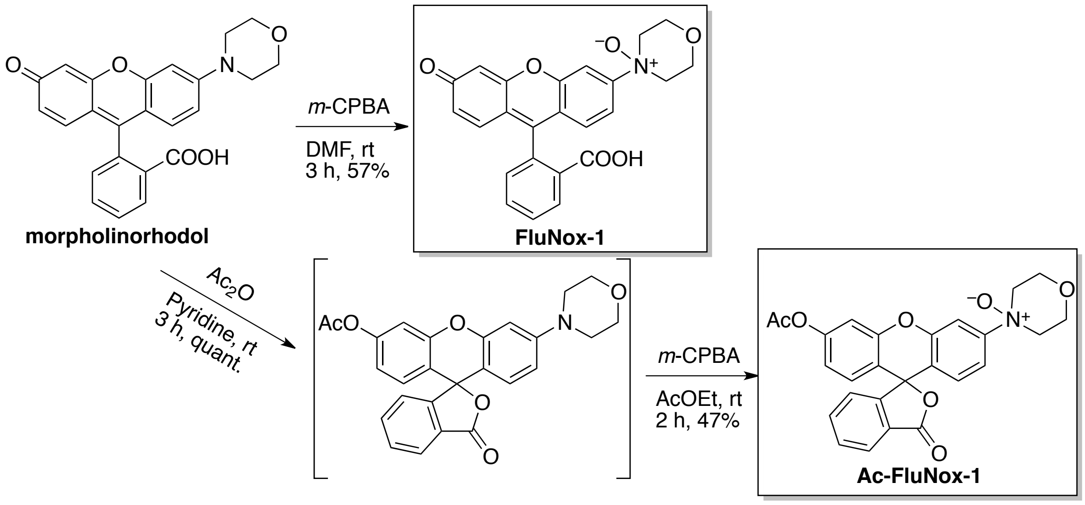

FluNox-1. Morpholinorhodol was prepared as previously described.[2] Morpholinorhodol (100 mg, 0.249 mmol) was dissolved in DMF (10 mL). After stirring at 0 °C for 10 min, *m*-CPBA (46.9 mg, 0.272 mmol) was added. The mixture was stirred at room temperature for 3 h. The mixture was evaporated, and the residue was purified by silica gel column chromatography ($CHCl_3$: MeOH = 6:1 to 4:1) to give FluNox-1 (59.1 mg, 57%). ^{1}H NMR (500 MHz, CD_3OD): δ 8.10 (d, J = 2.3 Hz, 1H), 8.05 (d, J = 8.0 Hz, 1H), 7.79 (t, J = 7.45, 1H), 7.73 (t, J = 7.45, 1H), 7.67 (dd, J = 9.2, 2.3 Hz, 1H), 7.2 (d, J = 7.4 Hz, 1H), 7.00 (d, J = 8.6 Hz, 1H), 6.75 (d, J = 2.3 Hz, 1H), 6.64 (d, J = 8.6 Hz, 1H), 6.59 (dd, J = 9.2 Hz, 2.3 Hz, 1H), 4.45 (t, J = 11.5 Hz, 2H), 4.18–4.12 (m, 2H), 3.94 (d, J = 12.6 Hz, 2H), 3.22 (m, 2H); ^{13}C NMR (125 MHz, CD_3OD+ 1% $CDCl_3$): δ 169.7, 160.1, 155.2, 152.8, 152.2, 151.8, 135.6, 130.2, 129.4, 128.9, 126.2, 124.8, 123.8, 120.8, 115.3, 113.0, 109.9, 109.2, 102.4, 82.3, 66.9, 61.6; HRMS (ESI+) *m/z* calcd for $[C_{24}H_{20}NO_6]^+$: 418.1285, found: 418.1304.

Ac-FluNox-1. To a solution of morpholinorhodol (150 mg, 0.374 mmol) in dry pyridine (10 mL), acetic anhydride (777 μL, 8.22 mmol) was added. The reaction mixture was stirred at room temperature for 3 hours. The mixture was evaporated, and the residue was dissolved by a mixed solution of AcOEt (10 mL) and toluene (5 mL), and the solution was washed with HCl (0.1 M, 10 mL). The organic layer was dried over $MgSO_4$, and evaporated to give the acetylated rhodol derivative quantitatively. This product was used for the next *N*-oxidation step without further purification. Then, the acetylated rhodol (130 mg, 0.29 mmol) was dissolved in ethyl acetate (10 mL), and *m*-CBPA (55.6 mg, 0.32 mmol) was added to the mixture at 0 °C. After stirring at room temperature for 2 h, additional portion of *m*-CPBA (55.6 mg, 0.32 mmol) was added. The reaction mixture was evaporated, and the residue was purified by silica gel column chromatography ($CHCl_3$: MeOH = 20: 1) to give Ac-FluNox-1 as a colorless solid. (62.2 mg, 47%).

^{1}H NMR (500 MHz, $CDCl_3$): δ 8.20 (d, J = 2.3 Hz, 1H), 8.05 (d, J = 7.4 Hz, 1H), 7.71 (t, J = 7.4 Hz, 1H), 7.67 (t, J = 7.4 Hz, 1H), 7.57 (dd, J = 8.6, 2.3 Hz, 1H), 7.18 (d, J = 7.4 Hz, 1H), 7.15 (s, 1H), 6.98 (d, J = 9.2 Hz,

1H), 6.86 (s, 2H), 4.70 (t, 2H, J = 11.5 Hz), 3.97–3.87 (m, 4H), 3.14 (d, 2H, J = 11.5 Hz), 2.32 (s, 3H); ^{13}C NMR (125 MHz, $CDCl_3$): δ 168.8, 168.7, 156.5, 152.5, 152.0, 151.22, 151.20, 135.4, 130.1, 128.9, 128.7, 125.6, 125.2, 123.7, 119.8, 118.0, 116.0, 115.5, 110.5, 110.4, 80.9, 67.5, 67.4, 62.0, 61.9, 20.9; HRMS (ESI+) *m/z* calcd for $[C_{26}H_{21}NO_7Na]^+$: 482.1216, found: 482.1237.

FluNox-2 and Ac-FluNox-2

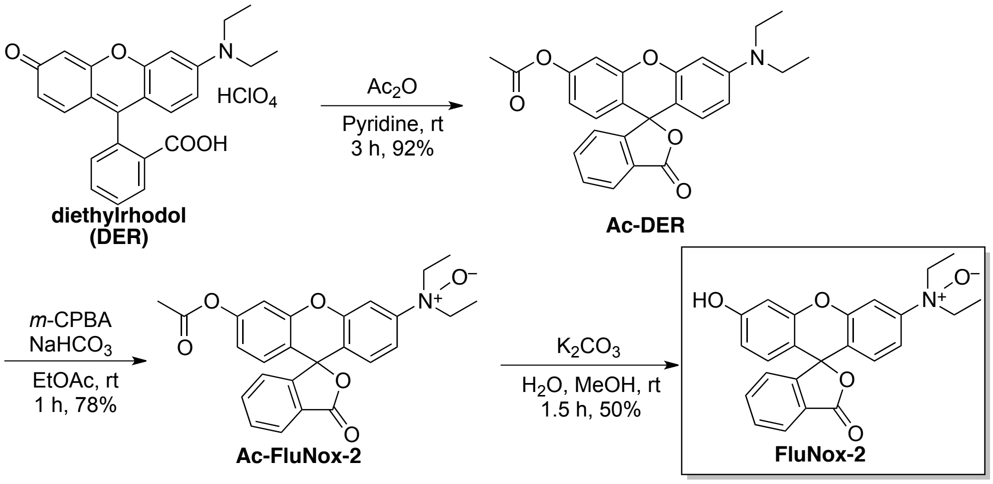

Ac-diethylrhodol (Ac-DER). To a solution of *N*,*N*-diethylrhodol (DER)[3] (204 mg, 0.42 mmol) in pyridine (5 mL) was added acetic anhydride (498 μL, 5.3 mmol). After stirring at room temperature for 3 h, the resulting mixture was evaporated. The residue was purified by silica gel column chromatography (EtOAc : Hexane = 1 : 5 to 1 : 3) to afford Ac-diethylrhodol as a pale pink solid (166 mg, 92%). ^{1}H NMR (500 MHz, $CDCl_3$): δ 8.01 (d, J = 7.4 Hz, 1H), 7.66 (m, 1H), 7.61 (m, 1H), 7.20 (d, J = 7.4 Hz, 1H), 7.04 (d, J = 2.3 Hz, 1H), 6.77 (d, J = 8.6 Hz, 1H), 6.74 (dd, J = 8.6 Hz, 2.3 Hz, 1H)), 6.57 (d, J = 9.2 Hz, 1H), 6.45 (d, J = 2.3 Hz, 1H), 6.36 (dd, J = 8.9 Hz, 2.6 Hz, 1H), 3.36 (q, J = 7.2 Hz, 4H), 2.31 (s, 3H), 1.17 (t, J = 7.2 Hz, 6H); ^{13}C NMR (125 MHz, $CDCl_3$): δ 168.5, 168.4, 153.0, 152.7, 152.3, 151.7, 149.7, 134.8, 129.6, 129.1, 128.8, 127.0, 124.9, 124.2, 117.2, 116.9, 110.1, 108.6, 104.8, 97.6, 83.7, 44.5, 21.2, 12.5; HRMS (ESI+): *m/z* calcd for $[C_{26}H_{24}NO_5]^+$: 430.1649, found : 430.1659.

Ac-FluNox-2. To a solution of Ac-DER (164 mg, 0.38 mmol) in EtOAc (5 mL) was added $NaHCO_3$ (35 mg, 0.42 mmol) and *m*-CPBA (104 mg, 0.42 mmol) at 0 °C. After stirring at room temperature for 1 h, the reaction mixture was evaporated. The residue was purified by silica gel column chromatography ($CHCl_3$: MeOH= 30 : 1 to 10 :1) to afford Ac-FluNox-2 as a pale purple powder (133 mg, 78%). ^{1}H NMR (400 MHz, $CDCl_3$): δ 8.06 (m, 2H), 7.73–7.64 (m, 2H), 7.29 (m, 1H), 7.20 (d, J = 7.2 Hz, 1H), 7.12 (s, 1H), 6.90 (d, J = 8.7 Hz, 1H), 6.85 (m, 2H), 3.71–3.63 (m, 4H), 2.33 (s, 3H), 1.18–1.14 (m, 6H); ^{13}C NMR (100 MHz, $CDCl_3$): δ 169.0, 168.9, 152.7, 152.2, 151.9, 151.5, 151.3, 135.5, 130.2, 128.9, 128.5, 126.0, 125.4, 123.9, 119.9, 118.1, 116.7, 116.3, 112.7, 110.6, 81.3, 67.2, 67.1, 21.1, 8.4, 8.3; HRMS (ESI+): *m/z* calcd for $[C_{26}H_{24}NO_6]^+$: 446.1598,

found : 446.1602.

FluNox-2. To a solution of Ac-FluNox-2 (61 mg, 0.14 mmol) in MeOH (3 mL) and H_2O (1 mL) was added K_2CO_3 (57 mg, 0.41 mmol). The mixture was stirred at room temperature for 1 h. Then, an additional portion of K_2CO_3 (57 mg, 0.41 mmol) was added to the reaction mixture. After stirring at room temperature for 0.5 h, the reaction mixture was neutralized by addition of 1 M HCl, and then evaporated. The residue was purified by silica gel column chromatography ($CHCl_3$: MeOH = 10 : 1) to afford FluNox-2 as a pale orange solid (27 mg, 50%). ^1H NMR (500 MHz, CD_3OD): δ 7.95 (d, *J* = 7.4 Hz, 1H), 7.82 (d, *J* = 2.3 Hz, 1H), 7.70 (m, 1H), 7.64 (m, 1H), 7.40 (dd, *J* = 8.9 Hz, 2.6 Hz, 1H), 7.15 (d, *J* = 8.0 Hz, 1H), 6.96 (d, *J* = 9.2 Hz, 1H), 6.66 (d, *J* = 2.3 Hz, 1H), 6.55 (d, *J* = 8.6 Hz, 1H), 6.51 (dd, *J* = 8.6 Hz, 2.3 Hz, 1H), 4.06–3.99 (m, 2H), 3.91–3.20 (m, 2H), 1.10–1.06 (m, 6H); ^{13}C NMR (125 MHz, CD_3OD): δ 170.8, 161.6, 153.9, 153.3, 153.2, 147.7, 136.9, 131.7, 131.1, 130.2, 127.5, 126.1, 125.2, 122.8, 117.3, 114.4, 112.5, 110.5, 103.7, 83.3, 67.8, 8.4; HRMS (ESI+): *m/z* calcd for $[C_{24}H_{22}NO_5]^+$: 404.1492, found : 404.1514.

SiRhoNox-1

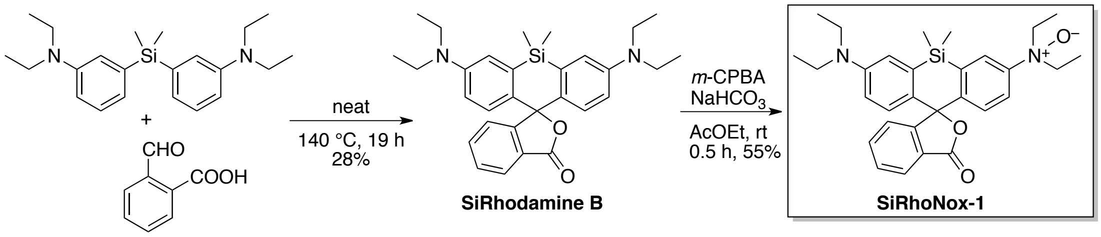

SiRhodamine B. SiRhodamine B was prepared according to a modified previous procedure.[4] In brief, to a 15 mL sealable pressure tube charged with a magnetic stir bar were added 3,3'-(dimethylsilanediyl) bis(*N*,*N*-diethylaniline) (654 mg, 1.8 mmol, 1.0 eq.) and 2-formylbenzoic acid (1.4 g, 9.2 mmol, 5.0 eq.). The tube was sealed tightly and heated at 140 °C for 19 h. After cooling to room temperature, the reaction mixture was dissolved in AcOEt (200 mL). The organic phase was washed with saturated $NaHCO_3$ aq. (50 mL × 2) and brine (50 mL), and dried over $MgSO_4$. After evaporation, the residue was purified by silica gel column chromatography (CH_2Cl_2: EtOAc = 100: 1) to give SiRhodamine B as a green solid (250 mg, 28%).
^1H NMR (500 MHz, $CDCl_3$): δ 7.96 (d, *J* = 7.4 Hz, 1H), 7.65 (t, *J* = 6.9 Hz, 1H), 7.55 (t, *J* = 7.4 Hz, 1H), 7.35 (d, *J* = 8.0 Hz, 1H), 6.91 (d, *J* = 2.9 Hz, 2H), 6.71 (d, *J* = 8.6 Hz, 2H), 6.47 (dd, *J* = 9.2, 2.9 Hz, 2H), 3.35 (q, *J* = 7.3 Hz, 8H), 1.15 (t, *J* = 7.2 Hz, 12H), 0.62 (s, 3H), 0.60 (s, 3H).

SiRhoNox-1. To a mixture of SiRhodamine B (300 mg, 0.62 mmol) and $NaHCO_3$ (104 mg, 1.2 mmol) in EtOAc (15 mL) was slowly added *m*-CPBA (182 mg, 0.74 mmol, 1.2 eq.) at 0 °C. The mixture was warmed to room temperature, and then stirred for 30 min. Insoluble materials were removed by filtration with a pad of celite, and then the filtrate was evaporated. The residue was purified with silica gel chromatography ($CHCl_3$:

MeOH = 15: 1) to give SiRhoNox-1 as an orange solid (172 mg, 55%).

^{1}H NMR (400 MHz, $CDCl_3$): δ 8.31 (d, J = 2.4 Hz, 1H), 8.01 (d, J = 7.7 Hz, 1H), 7.72 (t, J = 7.0 Hz, 1H), 7.61 (t, J = 7.5 Hz, 1H), 7.49–7.36 (m, 2H), 7.01 (d, J = 9.2 Hz, 1H), 6.94 (d, J = 2.9 Hz, 1H), 6.83 (d, J = 9.2 Hz, 1H), 6.51 (dd, J = 9.2, 2.9 Hz, 1H), 3.78-3.54 (m, 4H), 3.37 (q, J = 7.1 Hz, 4H), 1.21–1.02 (m, 12H), 0.70 (s, 3H), 0,67 (s, 3H); ^{13}C NMR (100 MHz, $CDCl_3$): δ 169.8, 152.5, 148.0, 146.5, 145.2, 138.4, 136.5, 133.5, 128.9, 128.1, 127.3, 126.6, 126.5, 125.8, 124.5, 121.7, 115.6, 112.1, 90.4, 66.5, 66.4, 43.9, 12.1, 8.0, −2.1; HRMS (ESI+): *m/z* calculated for $[C_{30}H_{37}N_2O_3Si]^+$: 501.2568, found 501.2571.

2. Steady-state absorption and fluorescence spectroscopy

The UV-vis absorption spectra were recorded on an Agilent 8453 photodiode array spectrometer equipped with a UNISOKU thermo-static cell holder (USP-203). Fluorescence spectra were recorded using a JASCO FP6600 with a slit width of 5 nm and 6 nm for excitation and emission, respectively. To reduce fluctuation in the excitation intensity during measurement, the lamp was kept on for 30 min prior to the experiment. The path length was 1 cm with a cell volume of 3.0 mL. Quantum yields were measured in 50 mM HEPES buffer (pH 7.4) by using a Quantaurus-QY absolute photo-luminescence quantum yields measurement system (C11347-01, Hamamatsu Photonics).

For determination of pK_a, absorption spectra were measured in 200 mM sodium phosphate buffer adjusted to various pH values. pH profiles at 450 nm (FluNox-1 and FluNox-2) and 575 nm (SiRhoNox-1) were fitted to the equation as follows:

$$A = \frac{A_0 + A_1 \times 10^{(\mathrm{pH}-\mathrm{p}K_a)}}{1 + 10^{(\mathrm{pH}-\mathrm{p}K_a)}}$$

where pK_a is the acid dissociation constants of FluNox derivatives: A_0 is the initial absorbance at 450 nm at pH 2.03, A_1 is the maximum absorbance at 450 nm associated with the corresponding pK_a values. The pK_a value for SiRhoNox-1 could not be calculated because of no pH dependent change in absorbance spectrum of SiRhoNox-1 (see Figure S3).

Fluorescence responses of the probes to various metal ions were measured as follows. An aqueous solution of transition metal ion species (stock solutions: 10 mM for $MnSO_4$, $CoSO_4$, $NiSO_4$, $FeSO_4$, $FeCl_3$, $CuSO_4$, and $ZnSO_4$; 100 mM for NaCl, KCl, $MgCl_2$, and $CaCl_2$) or $[Cu(MeCN)_4]PF_4$ (from 10 mM stock solution in MeCN) was added to give the final concentrations of 1 mM for Na^+, K^+, Mg^{2+}, and Ca^{2+} and 20 μM for other metal ion species. The mixtures were incubated for 1 h at room temperature, and then fluorescence spectra were measured. Stability against reductants and reactive oxygen species, and effect of chelator were tested under the conditions as follows.

$Na_2S_2O_3$: 100 μM from 100 mM stock solution in water

Sodium ascorbate : 1 mM from 100 mM stock solution in water

Cysteine : 1 mM from 100 mM stock solution in water

Glutathione : 1 mM from 100 mM stock solution in HEPES buffer (pH was adjusted to 7.4)

$NaNO_2$: 100 μM from 100 mM stock solution in water

$O_2^{\bullet-}$: 100 μM from saturated KO_2 solution in DMSO (ca. 1 mM)[5]

H_2O_2 : 100 μM from 100 mM stock solution in water

•OH : 200 μM H_2O_2 and 20 μM $FeSO_4$

NaOCl : 100 μM from 100 mM stock solution in water

NO : 100 μM NOC-5 from 10 mM stock solution in 0.1 M NaOH aq.

Fe^{2+} + Bpy : 20 µM $FeSO_4$ in the presence of 2,2'-bipyridyl (Bpy) from 100 mM stock solution in DMSO

Probe (2 μM) was incubated under each condition in 50 mM HEPES buffer (pH 7.4) for 1 h, and then fluorescence spectra were measured.

The stability test of SiRhoNox-1 against nitroreductase was performed by mixing 2 μM SiRhoNox-1 with 0.8 U/mL nitroreductase (from *Escherichia coli*, Sigma-Aldrich, N9274) in the presence of 200 μM β-NADPH in 50 mM HEPES buffer (pH 7.4). After incubation for 1 h at 37 °C, fluorescence intensities were acquired by fluorimeter as described above. The enzymatic activity (U) of the nitroreductase was determined from reaction rate of reduction of 5 μM cytochrome *c* (from horse heart, Nacalai Tesque 10429-84) in the presence of 200 μM β-NADPH at 37 °C in 50 mM HEPES buffer (pH 7.4).

3. Product analysis

To a solution of a probe (100 μM) in 50 mM HEPES buffer (pH 7.4) was added a solution of $FeSO_4$ (final, 1 mM). The mixture was kept for 1 h under an ambient condition. The products were analyzed with LC-MS system (LC-20AD, Shimadzu) equipped with a photodiode-array detector (SPD-M20A, Shimadzu) and an LCMS-IT-TOF mass spectrometer (Shimadzu) and with Waters X-Bridge C_{18} column (3.5 μm, 2.1 × 100 mm) eluted with gradient systems consisting of H_2O/CH_3CN as described in the caption of Figure S5. The retention times were compared with those of the parent dyes in 50 mM HEPES buffer (pH 7.4). Assignments of the compounds were based on the observed *m*/*z* values at each peak.

4. Cell culture experiments

Human hepatocellular carcinoma (HepG2) cells were cultured in modified essential medium (MEM, Gibco) containing 10% fetal bovine serum (FBS, Gibco), 1% Antibiotic-Antimycotic (ABAM, Gibco), and 2 mM glutamine at 37 °C in a 5% CO_2/ 95% air incubator. Two days before use, cells (1.0 × 10^5) were seeded on Advanced TC glass-bottomed dishes (CELLview™ Cell Culture Dish, Greiner).

5. Confocal fluorescence imaging experiments

Confocal fluorescence images were acquired with Zeiss LSM700 laser confocal microscopy or Olympus IX83 microscope equipped with a 130 W mercury lump, an EMCCD camera (Hamamatsu Photonics, ImagEM), and a disk scan confocal unit (DSU). Images were obtained with appropriate filter sets for each dye as follows. CoNox-1: coumarin filter set (excitation = 370–410 nm, emission = 460–490 nm, dichroic mirror = 405 nm). FluNox-1: FITC filter set (excitation = 465–500 nm, emission = 516–556 nm, dichroic mirror = 495 nm) SiRhoNox-1: Cy5 filter set (excitation = 610–650 nm, emission = 672–712 nm, dichroic mirror = 660 nm) For all imaging experiments, Hank's Balanced Salt Solution (HBSS, Gibco) containing calcium and magnesium without phenol red was used. For Fe^{2+} uptake experiments, cells were treated with 100 μM ferrous ammonium sulfate (FAS, $Fe(NH_4)_2(SO_4)_2$ from 10 mM stock solution in water) in MEM without FBS at 37 °C for 30 min. For all imaging experiments, after washing the cells with HBSS (×3), CoNox-1, Ac-FluNox-1, or SiRhoNox-1 (5 μM, from 1 mM stock solution in DMSO) was added. After incubation for 2 h (for CoNox-1) or 1 h (for Ac-FluNox-1 and SiRhoNox-1) at 37 °C, the cells were washed with HBSS (×1), and then imaged. For chelation experiments, 1 mM 2,2'-bipyridyl (Bpy, from 100 mM stock solution in DMSO) was added to the cells simultaneously with the probes.

For co-staining experiments, 500 nM ER-Tracker™ Red was used with 5 μM CoNox-1 in HBSS for 2 h after treatment of the cells with 50 μM FAS in the presence of 20 μM pyrithione in MEM (without FBS). For co-staining with SiRhoNox-1, the cells were incubated with 5 μM SiRhoNox-1 with 500 nM ER-Tracker™ Green in HBSS for 30 min at 37 °C after treatment of the cells with 100 μM FAS. For all the control experiments, the corresponding amounts of vehicles (FAS: water, Bpy: DMSO) were added to the cells instead. Image analysis was performed with ImageJ.

6. Imaging experiments under hypoxia

Hypoxia treatments were performed in a hypoxia chamber (InvivO2 hypoxia workstation, Baker Ruskinn, USA) under control of oxygen concentration (1% or 5%). All the mediums and solvents used for hypoxic treatments were pre-incubated for more than 8 h prior to use in the chamber, and all the treatments with SiRhoNox-1, Bpy, and DPI were performed in the hypoxia chamber. HepG2 cells (8×10^4) were seeded on each well of Advanced-TC glass bottom dishes. After incubation in MEM (+10% FBS, 2 mM glutamine, and 1% ABAM) for 2 days prior to use, the cells were placed in the hypoxia chamber and incubated for 2, 4, 8, or 12 h under the controlled oxygen concentration of 1% or 5% at 37 °C. For the cell-staining experiments, the cells were washed with HBSS, and then the medium was replaced with new HBSS solution containing 5 μM SiRhoNox-1with or without 1 mM Bpy or DPI (10 or 100 μM, diphenyliodonium chloride). After incubation of the cells in the chamber for 30 min, the cells were washed with HBSS and then imaged. For control experiments (20% O_2), the cells were incubated in an incubator (37 °C, 5% CO_2, 95% air) for the same period, and all the treatments were performed in a standard clean bench.

7. Quantification of total intracellular iron by atomic absorption spectrometry

HepG2 cells (1.2×10^5 cells) were seeded on a 10 cm dish 2 days prior to use. The cells were cultured under hypoxia (1% O_2) as described above for 0, 2, 4, 8, and 12 h. Then, the medium was washed with cold phosphate-buffered saline (PBS, 3 mL × 3). The cells were removed from the dishes by using a scraper, and then the suspension was centrifuged (1,000 rpm, 5 min). The supernatant was carefully removed, and the cells were re-suspended into conc. HNO_3 (100 μL). The suspension was heated to 90 °C for 4 h to dissolve the cell bodies. The lysate was diluted to 2 mL with distilled water. Concentrations of iron in the samples were measured by furnace atomic absorption spectroscopy with a Shimadzu AA-7000 atomic absorption spectrometer. The obtained values (ng/mL) were normalized with the cell numbers (per 10^6 cells).

Total 4 dishes were prepared for each experiment as described above. 3 dishes were used for iron quantification, and the rest was used to calculate the number of cells. To determine the cell numbers, the cells were collected by trypsinization at the same time point with the other 3 dishes, and the number of the cells was counted by a hematocytometer.

8. Western blotting of HIF-1α and ferritin

HepG2 cells (6.0×10^6 cells) were seeded on a 10 cm dish and then incubated at 37 °C for 12 h. The cells were cultured under aerobic or hypoxic (20% or 1% O_2) conditions for 2, 4, 8, or 12 h. Then, the cells were collected by cell scraper and lysed by RIPA (radio-immunoprecipitation assay) buffer for 30 min on ice. After centrifugation (14,000 rpm, 4 °C, 10 min) of the cell lysate, the supernatants were applied to SDS-PAGE (10% SDS). Protein concentrations were determined by BCA-assay according to the manufacturer's protocol (ThermoFisher Scientific). The cell extracts (total 100 μg/mL of proteins) were subjected to SDS-PAGE (10% SDS) followed by electrotransfer to a nitrocellulose membrane. Immuno-blotting was performed by using primary antibodies for HIF-1α (1:1000, Sigma-Aldrich), ferritin (1:1000, ab75973, Abcam), and for β-actin (1:3000, Sigma-Aldrich) and secondary antibodies of horseradish peroxidase-conjugated anti-mouse IgG (for HIF-1α, 1:1000, Sigma-Aldrich), anti-rabbit IgG (for ferritin, 1:2000, sc2004, Santa Cruz Biotechnology), and anti-goat IgG (for β-actin, 1:3000, Sigma-Aldrich). The proteins were visualized by Immobilon™ Western Chemiluminescent HRP substrate (Pierce) and measured on a luminescent image analyzer (LAS 3000, Fujifilm).

9. Preparation and imaging study of spheroids[6]

HepG2 cells (1.0×10^3 cells/well) were seeded on a PrimeSurface® 96-well plate (Sumitomo Bakelite Co. Ltd., Japan). After incubation for 5 days in MEM (+10% FBS), the spheroids growing to 500 μm of diameter were picked up and transferred to a new PrimeSruface® 96-well plate. Then the spheroids were treated with 10 μM SiRhoNox-1 or 200 μM pimonidazole hydrochloride (Hypoxyprobe, Inc., USA) in MEM (−FBS, 0.5% DMSO) at 37 °C for 2 h. The medium was exchanged to fresh medium (MEM, −FBS), and the spheroids were

incubated for further 30 min. Then, the spheroids were fixed by treatment with a mixture of formalin (10%) and sucrose (10%) in water at room temperature for 2 h. After washing with PBS, the spheroids were embedded into optimal cutting temperature (O.C.T.) compound (Sakura Finetek Japan) and frozen at −20 °C overnight. The embedded spheroids were cut into 7 μm thick slices with a cryostat (Leica). The sections placed on slide glasses without holes in the central area were selected for next step.

For imaging SiRhoNox-1-treated spheroids, the sections were further stained with 0.05% (w/v) DAPI (nuclear staining dye) in 7% FBS/PBS solution at room temperature for 20 min. After washing with PBS (× 5), they were mounted with coverslips using VECTASHIELD mounting medium (Vector Laboratories, USA) as the manufacturer's protocol.

For immuno-staining of pimonidazole, the sections were treated with a solution of 0.5% Triton X-100 in PBS at room temperature for 30 min, washed by PBS twice, incubation in 7% FBS/PBS blocking solution at room temperature for 30 min, and then stained with 2% FITC MAb1 (Hypoxyprobe, Inc., USA), a fluorescein-conjugated antibody for pimonidazole-thiol adducts, in 7% FBS/PBS at room temperature for 1 h. After washing with PBS, the slices were further stained with 0.05% (w/v) DAPI in 7%FBS/PBS solution at room temperature for 20 min, washed with PBS (× 5), and mounted with coverslips using VECTASHIELD mounting medium.

The prepared sections were observed with the confocal microscope (Olympus IX83) with appropriate filters.

<Figures>

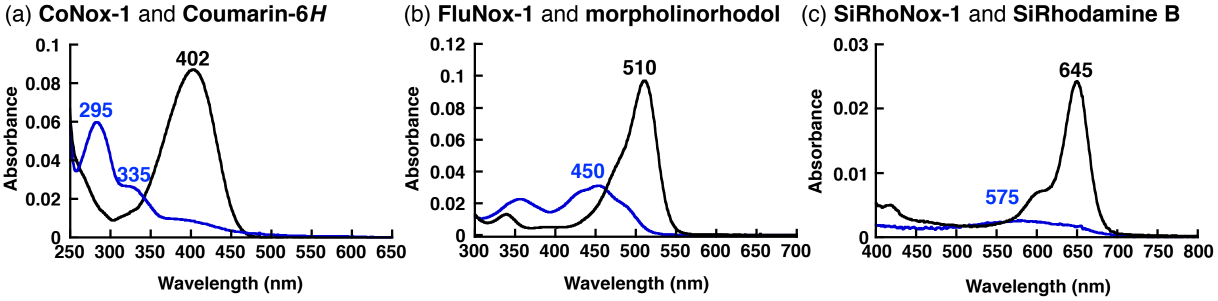

Figure S1 Absorption spectra of the *N*-oxide probes (blue lines) and their corresponding fluorophores (black lines). (a) CoNox-1 and coumarin-6*H*. (b) FluNox-1 and morpholinorhodol. (c) SiRhoNox-1 and SiRhodamine B. All the spectra were acquired with 2 μM probes or dyes in 50 mM HEPES buffer (pH 7.4. 0.2% DMSO as cosolvent).

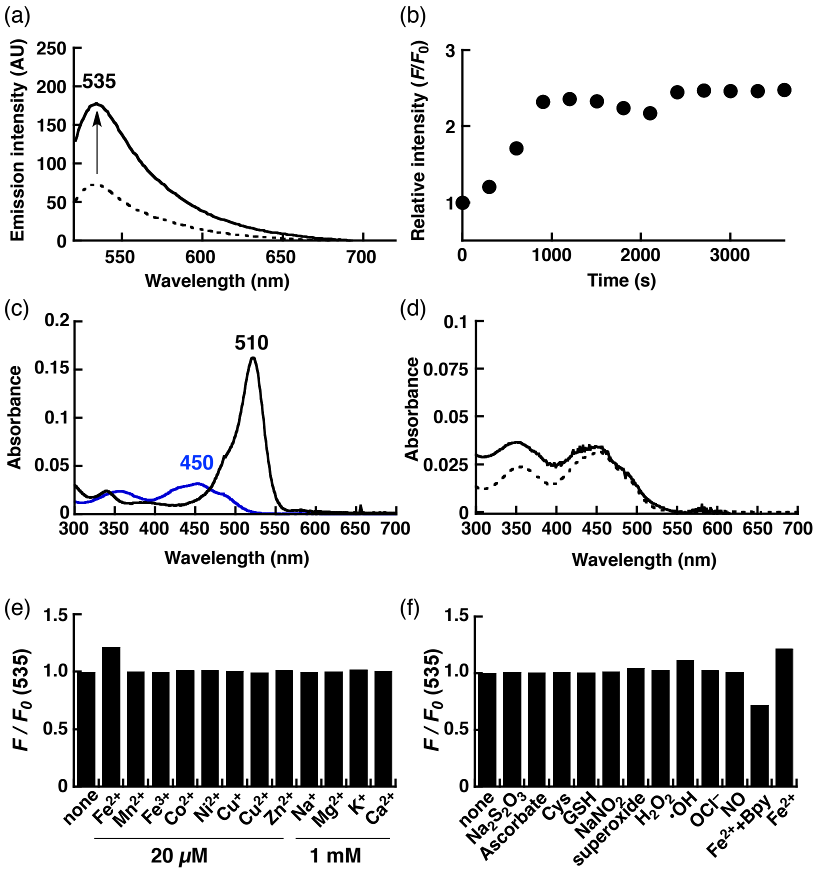

Figure S2. Photophysical measurements of FluNox-2. (a) Fluorescence response of FluNox-2 against Fe^{2+}. (b) Plot of response kinetics of FluNox-2. Relative fluorescence intensity is plotted every 300 sec. (c) Absorption spectra of FluNox-2 (blue line) and diethylrhodol (black line). (d) Absorption spectral change of FluNox-2 upon addition of Fe^{2+}. (e) Fluorescence response of FluNox-2 against various metal ions. (1 mM for alkali and alkaline earth metal ions, and 20 μM for all other metal ions). (f) Fluorescence response of FluNox-2 against various reductants and reactive oxygen species. All the data was collected by using FluNox-2 as a probe and otherwise with the same conditions with those of FluNox-1.

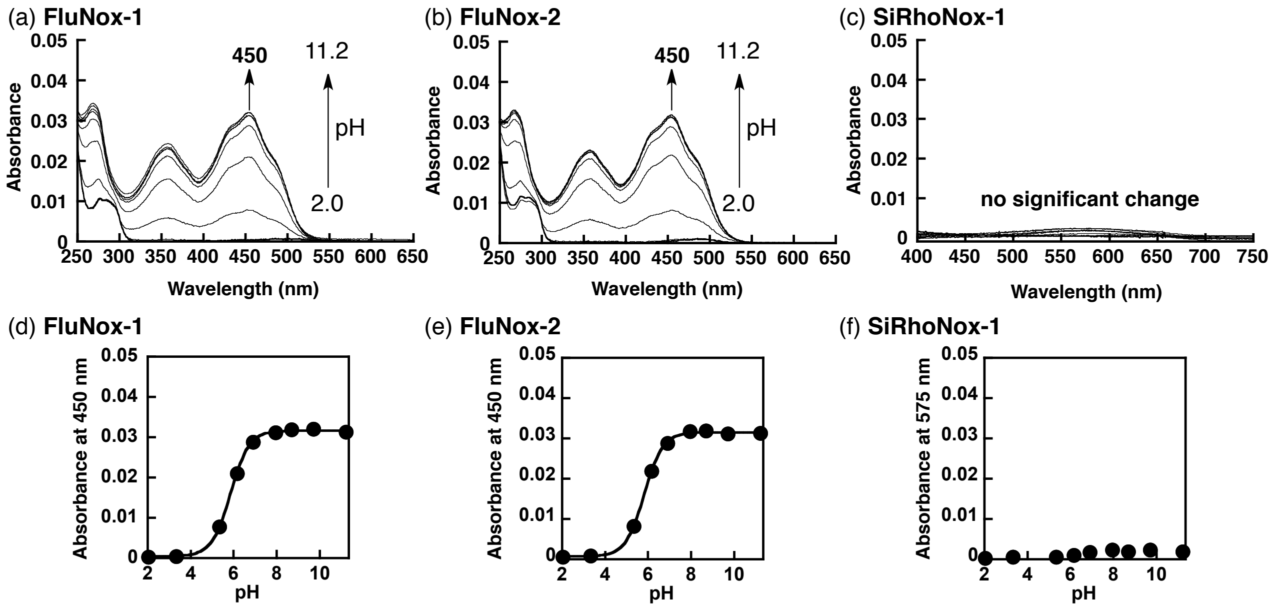

Figure S3. Absorption spectral changes of (a) FluNox-1, (b) FluNox-2, and (c) SiRhoNox-1 over various pH (2.03, 3.31, 5.32, 6.16, 6.89, 7.94, 8.68, 9.69, and 11.2) in 200 mM phosphate buffer. (d, e, f) Plot of absorbance at 450 nm (d,e, FluNox-1 and FluNox-2) 575 nm (f, SiRhoNox-1) against pH. The pK_a values calculated from the pH profiles were 5.80 (FluNox-1) and 5.82 (FluNox-2).

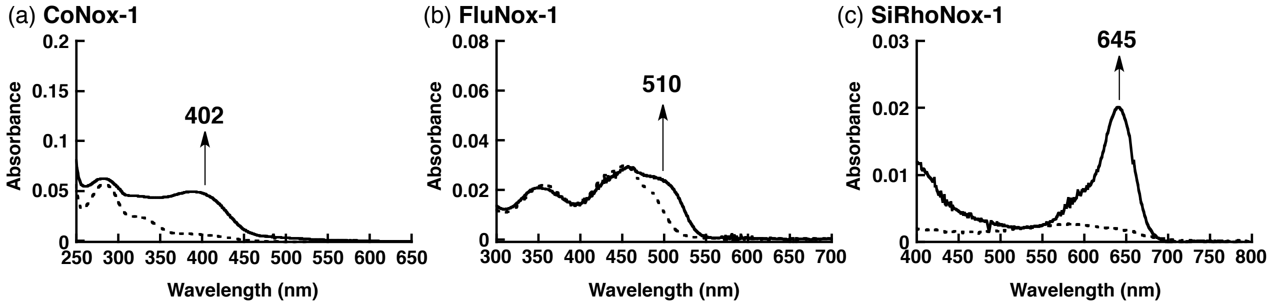

Figure S4 Absorption spectral changes of (a) CoNox-1, (b) FluNox-1, and (c) SiRhoNox-1 upon addition of $FeSO_4$. Dotted lines and solid lines indicate before and after incubation for 1 h, respectively. All the spectra were acquired with 2 μM probes and 20 μM $FeSO_4$ in 50 mM HEPES buffer (pH 7.4. 0.2% DMSO as cosolvent).

.

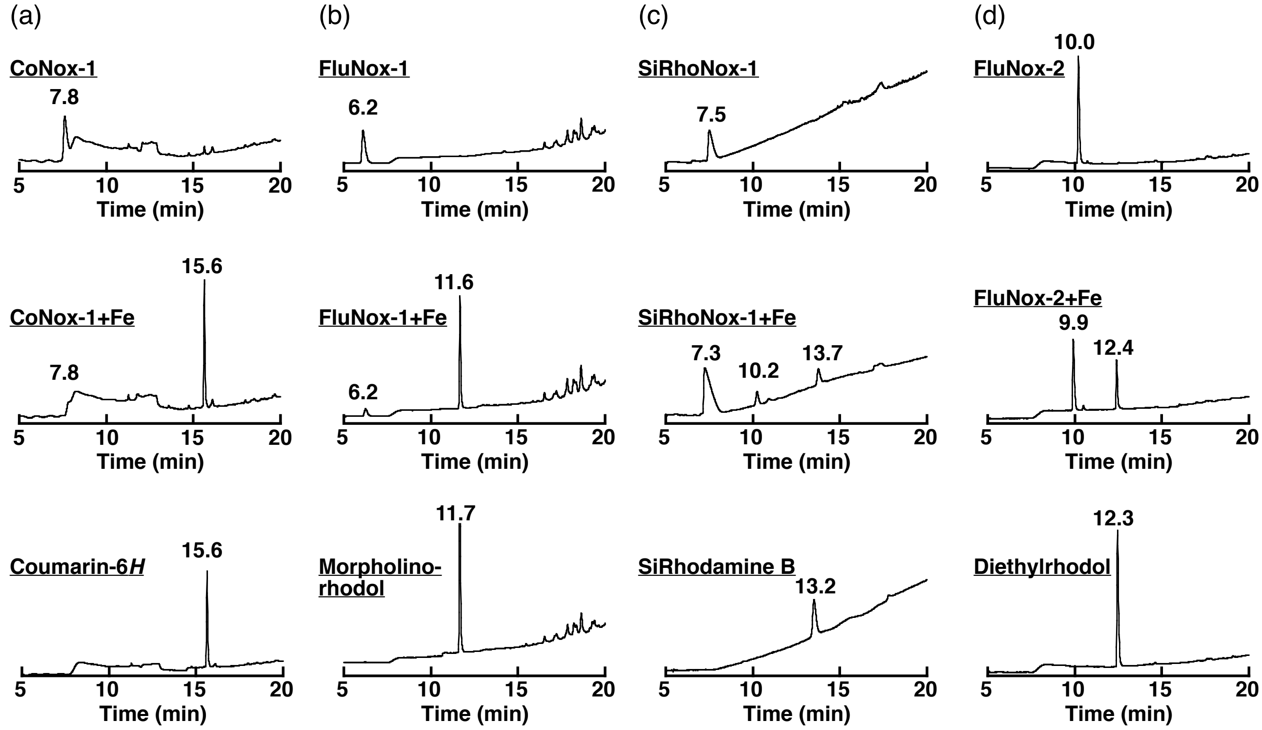

Figure S5. HPLC-IT-TOF mass monitoring of the reaction between the probes and Fe^{2+}. (a) CoNox-1, (b) FluNox-1, (c) SiRhoNox-1, and (d) FluNox-2. Each probe (100 μM) was incubated with 1 mM Fe^{2+} for 1 h in 50 mM HEPES buffer (pH 7.4). $Fe(NH_4)_2(SO_4)_2$•$6H_2O$ was used as Fe^{2+} source. The parent fluorophores, Coumarin-6*H*, Morpholinorhodol, SiRhodamine B, and diethylrhodol were used as the authentic samples. The reaction mixtures were analyzed by HPLC system equipped with a photodiode-array detector and an IT-TOF-MS detector. The gradient systems using H_2O (solvent A) and MeCN (solvent B) containing 0.05 % formic acid were as follows. Data collection by monitoring absorption at 256 nm and mass spectra was started at 5 min after eluting highly polar components such as iron salt and HEPES with the initial solvents, and the gradients started then.

(a) CoNox-1: 5%B to 95%B over 15 min.
(b) FluNox-1: 20%B to 95%B over 15 min.
(c) SiRhoNox-1: 50%B to 95%B over 15 min.
(d) FluNox-2: 20%B to 90%B over 15min.

Total ion mass spectra provided mass peaks at each time point as follows.

(a) 7.8 min: 258.1 (calcd for $[\text{CoNox-1+H}]^+$ = 258.1), 15.6 min: 242.1 (calcd for [coumarin-6*H*+H]$^+$ = 242.1).
(b) 6.2 min: 418.1 (calcd for $[\text{FluNox-1+H}]^+$ = 418.1), 11.6 min, 11.7 min: 402.1 (calcld for [morpholinorhodol+H]$^+$ = 402.1)
(c) 7.3 min, 7.5 min: 501.2 (calcd for $[\text{SiRhoNox-1+H}]^+$ = 501.1), 10.2 min: 457.2 (calcd for [SiRhodamine B (ethyl group cleaved) +H]$^+$ = 457.2), 13.2 and 13.7 min: 485.3 (calcd for [SiRhodamine B+H]+ = 485.3).
(d) 9.9 and 10.0 min: 404.1 (calcd for $[\text{FluNox-2+H}]^+$ = 404.1), 12.3 min, 12.4 min: 388.1 (calcd for [diethylrhodol+H]$^+$ = 388.1)

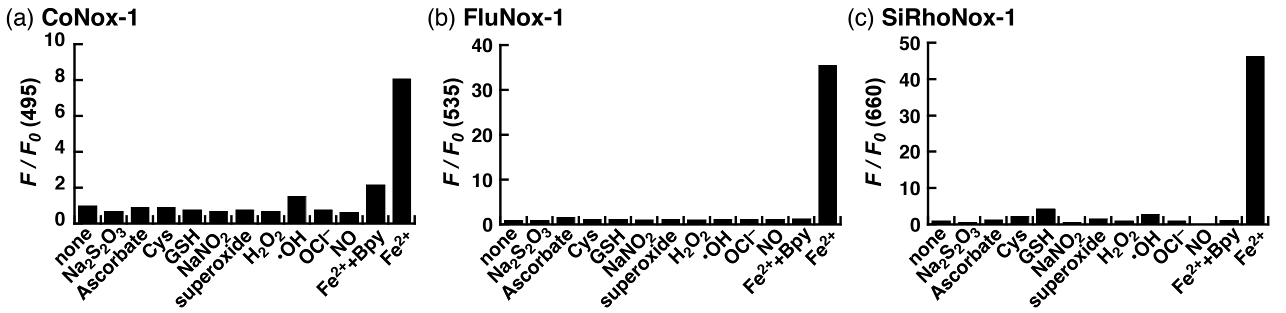

Figure S6. Fluorescence response of (a) CoNox-1, (b) FluNox-1, and (c) SiRhoNox-1 against various reductants, reactive oxygen species. Bars represent relative fluorescence intensities at 495 nm (a), 535 nm (b), and 660 nm (c). λ_{ex} = 405 nm for (a), 488 nm for (b), and 630 nm for (c). All data were collected with 2 μM probe after 1 h incubation in 50 mM HEPES buffer (pH 7.4).

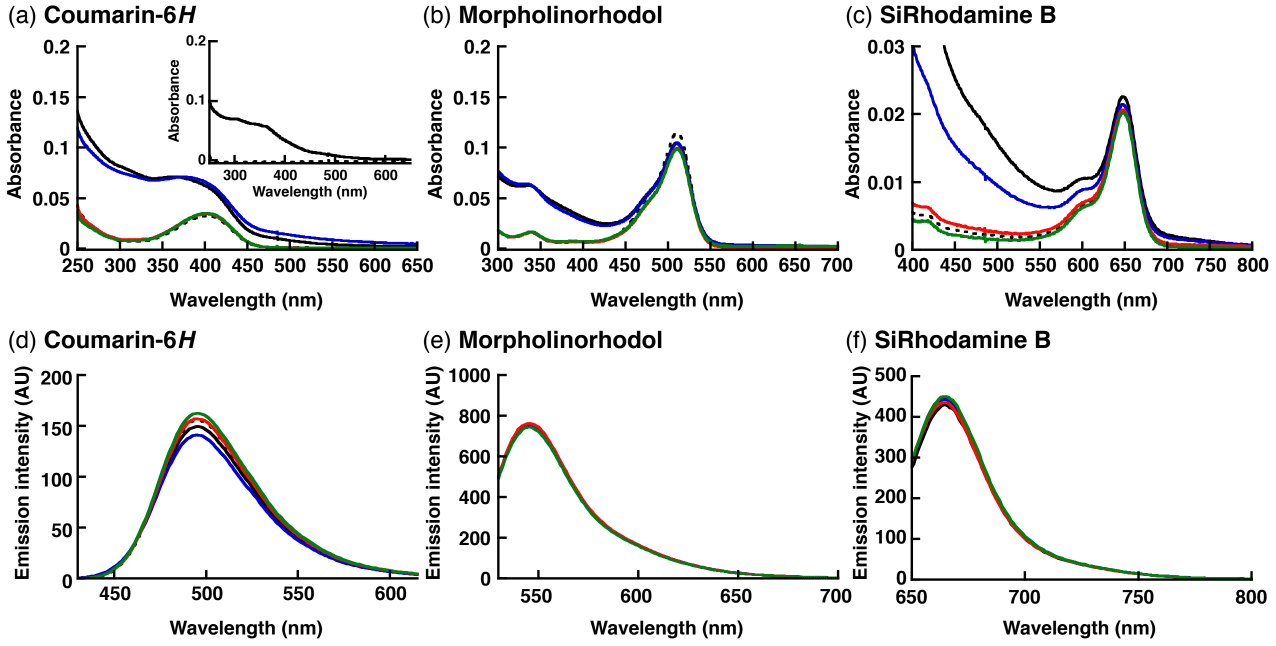

Figure S7 Stability test of the parent fluorophores (2 μM), coumarin-6*H* (a,d), morpholinorhodol (b,e), and SiRhodamine B (c,f) by absorption (a–c) and fluorescence spectroscopy (d–f) in the absence (dashed black lines), the presence of Fe^{2+} (20 μM, solid black lines), •OH (generated with 20 μM Fe^{2+} + 200 μM H_2O_2, blue lines), H_2O_2 (200 μM, red lines), or NaOCl (200 μM, green lines). The spectra were acquired after incubation for 1 h in 50 mM HEPES buffer (pH 7.4, 0.2%DMF as co-solvent). Excitations were provided by 405 nm (d), 488 nm (e), and 630 nm (f). Inset: Solid line: Absorption spectrum of 20 μM $FeSO_4$ in 50 mM HEPES buffer (pH 7.4, 0.2% DMF). Dashed line: Blank (50 mM HEPES buffer, pH 7.4, 0.2% DMF) The broad absorption around 330 nm (black and blue lines in (a), (b), and (c)) was attributed to Fe^{3+} species, which generates through auto-oxidation of Fe^{2+} in aqueous buffer under ambient condition.

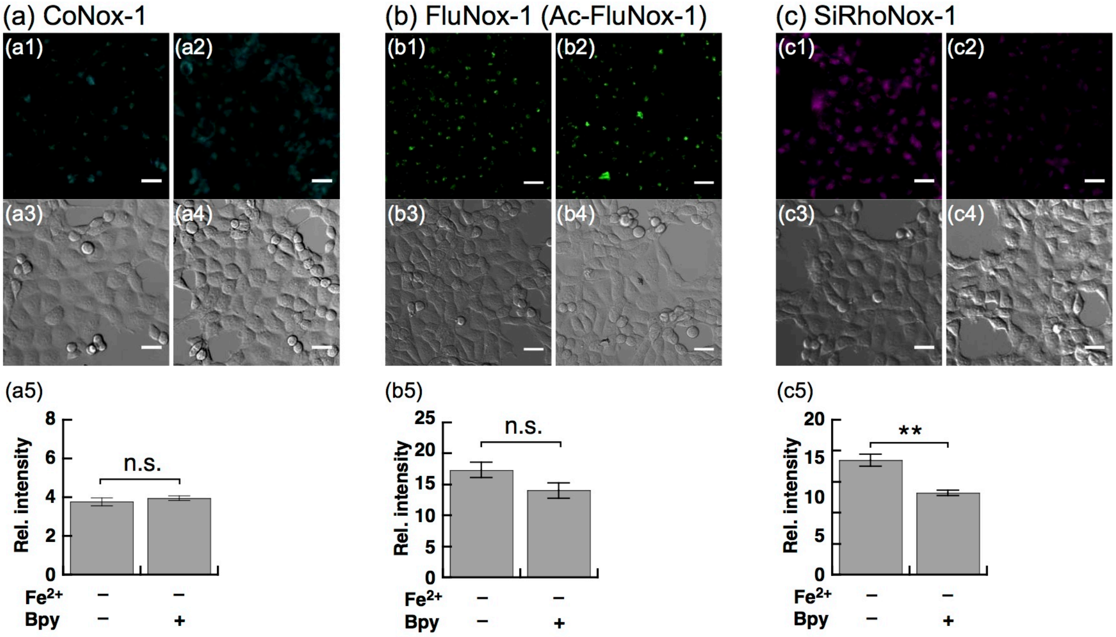

Figure S8. Confocal fluorescence microscopy analysis of endogenous labile Fe^{2+} in HepG2 cells by using (a) CoNox-1, (b) FluNox-1, and (c) SiRhoNox-1. (1) Images of the cells treated with 5 μM probe for 1 h. (2) Images of the cells treated with probe in the presence of 1 mM Bpy. (3,4) Images of the same slices of (1) and (2). (5) Quantification of data in (1) and (2). Statistical analyses were performed with a Student's *t*-test. $^{**}P < 0.001$ (n = 3). Error bars mean ± s.e.m. "n.s." means not significant. Scale bars indicate 25 μm.

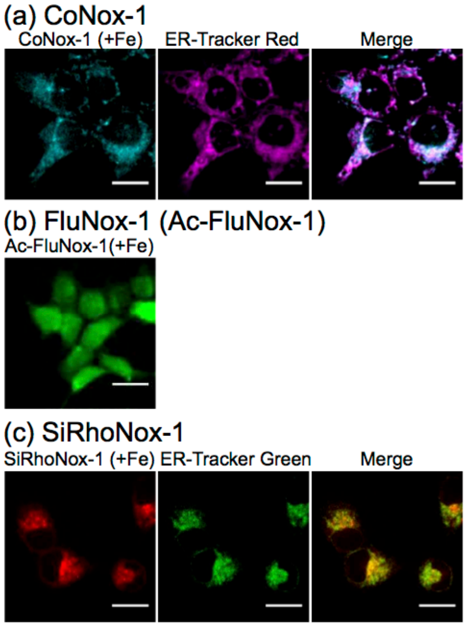

Figure S9. Confocal fluorescence images to verify intracellular localization of the probes. (a) Co-staining of cells with CoNox-1 and ER-tracker Red in the presence of 50 μM Fe^{2+} and 20 μM pyrithione as an ionophore for Fe^{2+}. (b) Staining with Ac-FluNox-1. (c) Co-staining of cells with SiRhoNox-1 and ER-tracker Green. All the staining experiments were done after treatment of the cells with 100 μM FAS for 30 min. Scale bars indicate 25 μm.

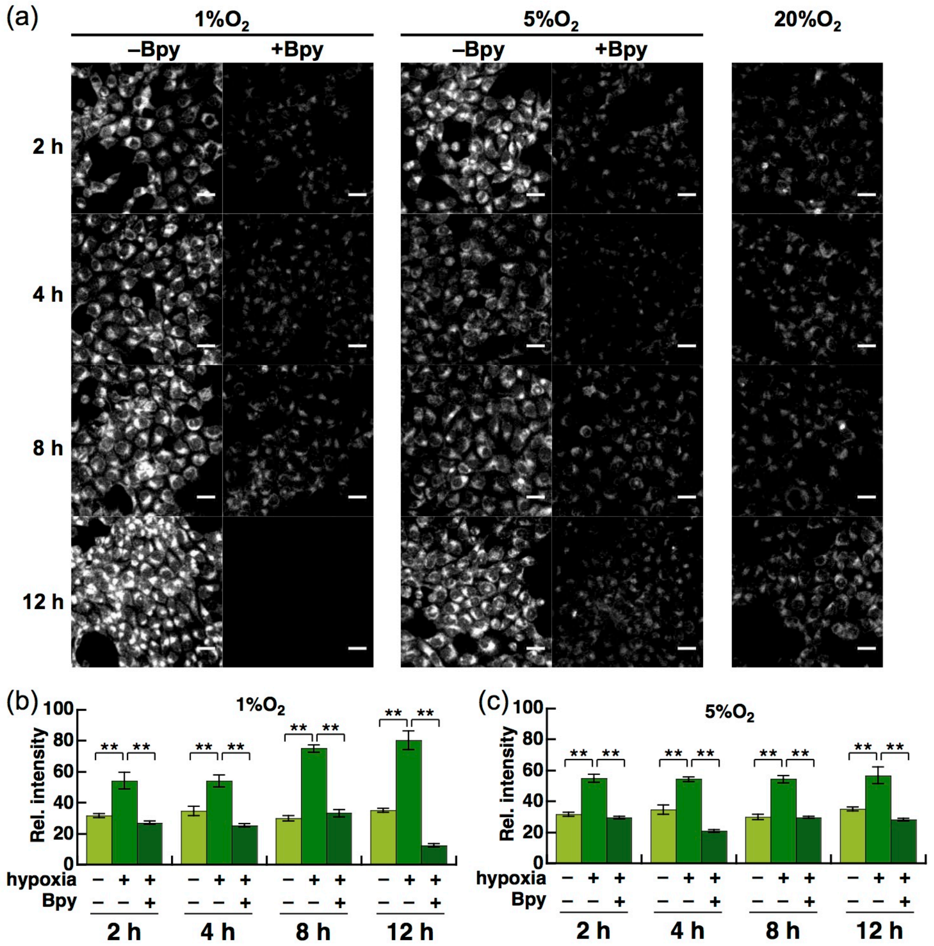

Figure S10. Confocal fluorescence microscopy analyses of HepG2 cells incubated under various concentration of oxygen (1%, 5%, and 20%) for various time (2 h, 4 h, 8 h, and 12 h) and stained by 5 μM SiRhoNox-1 in the presence or absence of 1 mM 2,2'-bipyridyl (Bpy) for 30 min. (a) Representative images of each condition. (b) Quantification of images taken under 1% oxygen (hypoxia+) and 20% oxygen (hypoxia−) (n = 4). (c) Quantification of images taken under 5% oxygen (hypoxia+) and 20% oxygen (hypoxia−) (n = 4). All the images were taken by using a Cy5 filter set as described above. Scale bars indicate 25 μm. Statistical analyses were performed with a Student's *t*-test. ***P* < 0.01 (n = 4). Error bars mean ± s.e.m.

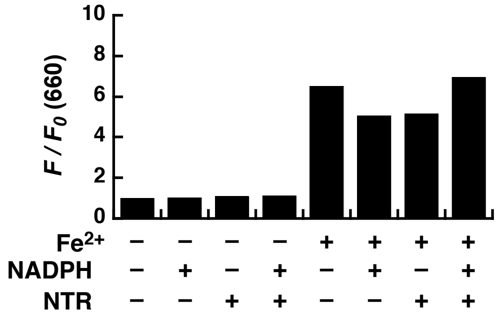

Figure S11. Fluorescence response of 2 μM SiRhoNox-1 against nitroreductase (0.8 U/mL) in the presence or absence of 200 μM β-NADPH. All the data were acquired after incubation at 37 °C for 1 h in 50 mM HEPES buffer (pH 7.4).

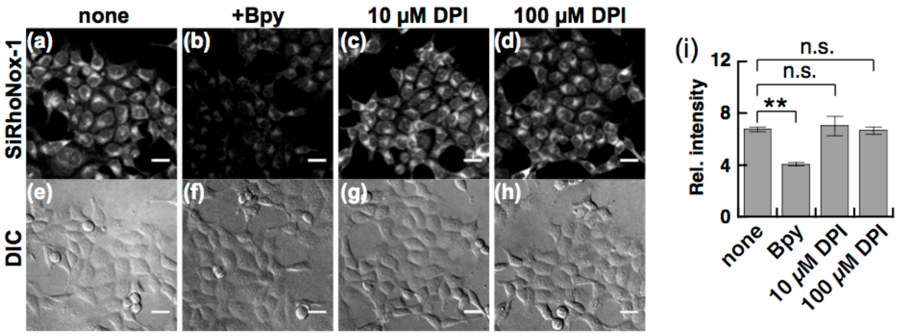

Figure S12. Confocal fluorescence microscopy analyses for evaluation of effect of diphenyliodonium chloride (DPI) on fluorescence response of SiRhoNox-1 under hypoxic condition (1% O_2). (a) HepG2 cells stained with 5 μM SiRhoNox-1. (b) HepG2 cells stained with 5 μM SiRhoNox-1 in the presence of 1 mM Bpy. (c) HepG2 cells stained with 5 μM SiRhoNox-1 in the presence of 10 μM DPI. (d) HepG2 cells stained wit 5 μM SiRhoNox-1 in the presence of 100 μM DPI. (e–h) DIC images for the same slices of (a–d). (i) Statistical analyses were performed with a Student's *t*-test. $^{**}P < 0.01$ (n = 4). Error bars mean ± s.e.m. "n.s." means not significant. Scale bars indicate 25 μm. All the cells were incubated under 1% O_2 for 8 h.

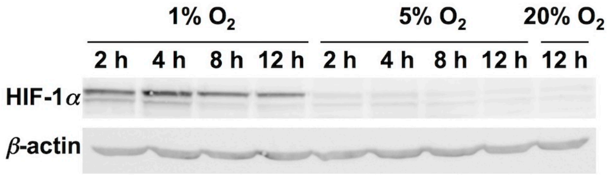

Figure S13. Western blot analyses of HIF-1α in HepG2 cells incubated under 1%, 5%, or 20% O_2 conditions for 2, 4, 8, and 12 h.

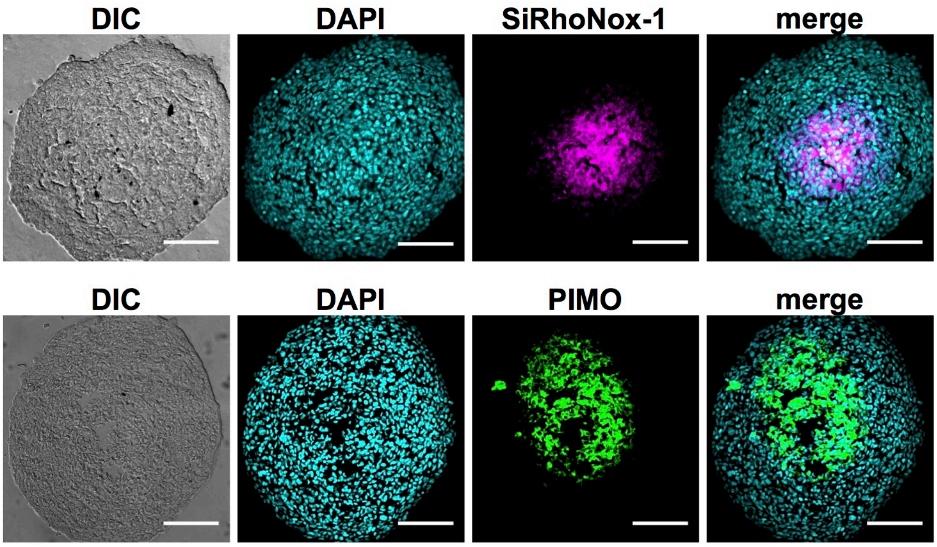

Figure S14 Confocal fluorescence images of slices of spheroids of HepG2 cells. The spheroids were fixed, embedded, and sectioned prior to imaging. Upper: Images of a spheroid stained with SiRhoNox-1 and DAPI (nucleus). Bottom: Images of a pimonidazole-treated spheroid stained with anti-pimonidazole antibody and DAPI. Scale bars indicate 100 μm.

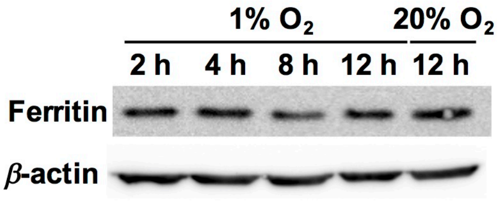

Figure S15. Western blot analyses of ferritin in HepG2 cells incubated under 1% or 20% O_2 conditions for 2, 4, 8, and 12 h.

<References>

1 L. Yuan, W. Lin, J. Song and Y. Yang, *Chem. Commun.*, 2011, **47**, 12691–12693.

2 T. Peng and D. Yang, *Org. Lett.*, 2010, **12**, 496–499.

3 M. Kamiya, D. Asanuma, E. Kuranaga, A. Takeishi, M. Sakabe, M. Miura, T. Nagano and Y. Urano, *J. Am. Chem. Soc.*, 2011, **133**, 12960–12963.

4 B. Wang, X. Chai, W. Zhu, T. Wang and Q. Wu, *Chem. Commun.*, 2014, **50**, 14374–14377.

5 A. R. Lippert, E. J. New and C. J. Chang, *J. Am. Chem. Soc.*, 2011, **133**, 10078–10080.

6 N. Nozawa-Suzuki, H. Nagasawa, K. Ohnishi and K. Morishige, *Biochem. Biophys. Res. Commun*., 2015, **457**, 706-711.

^{1}H- and ^{13}C-NMR spectra of newly synthesized compounds

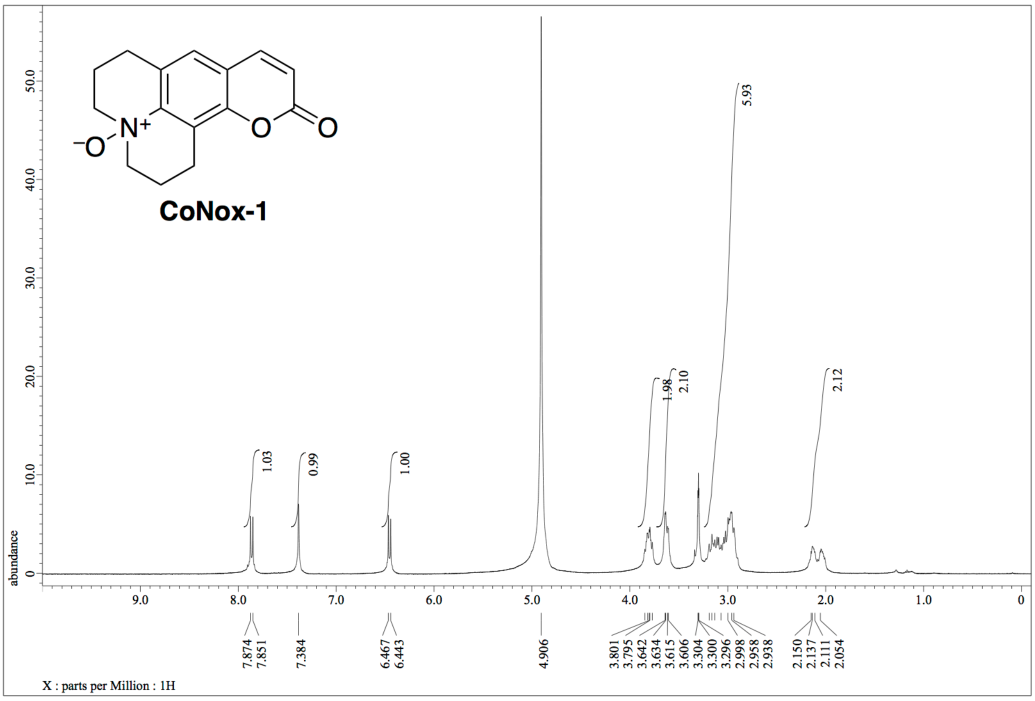

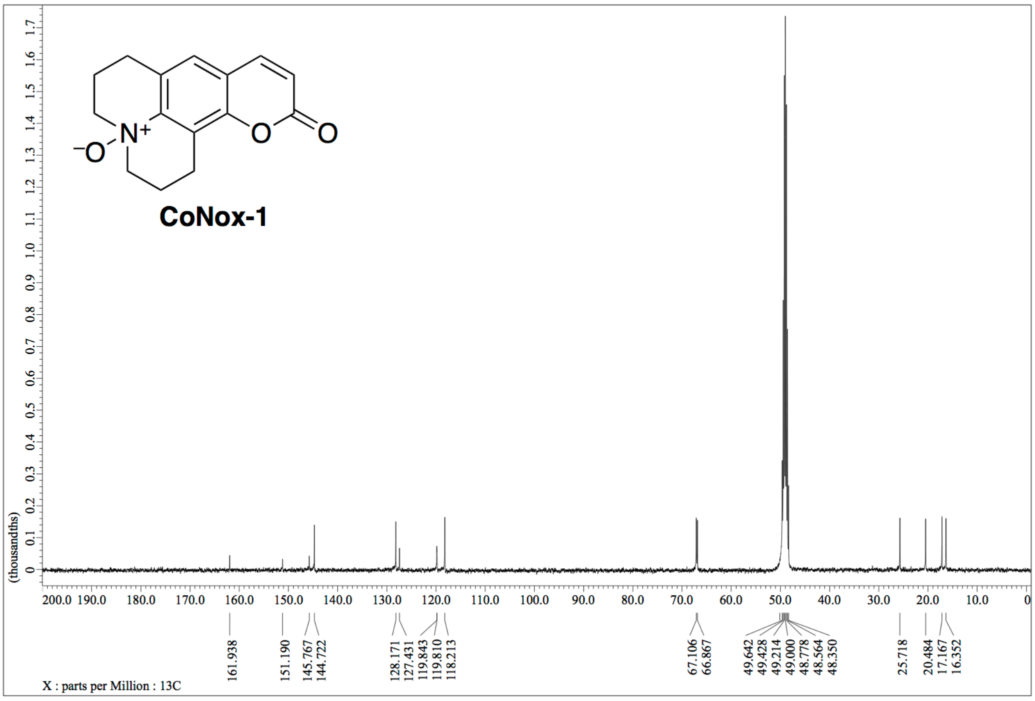

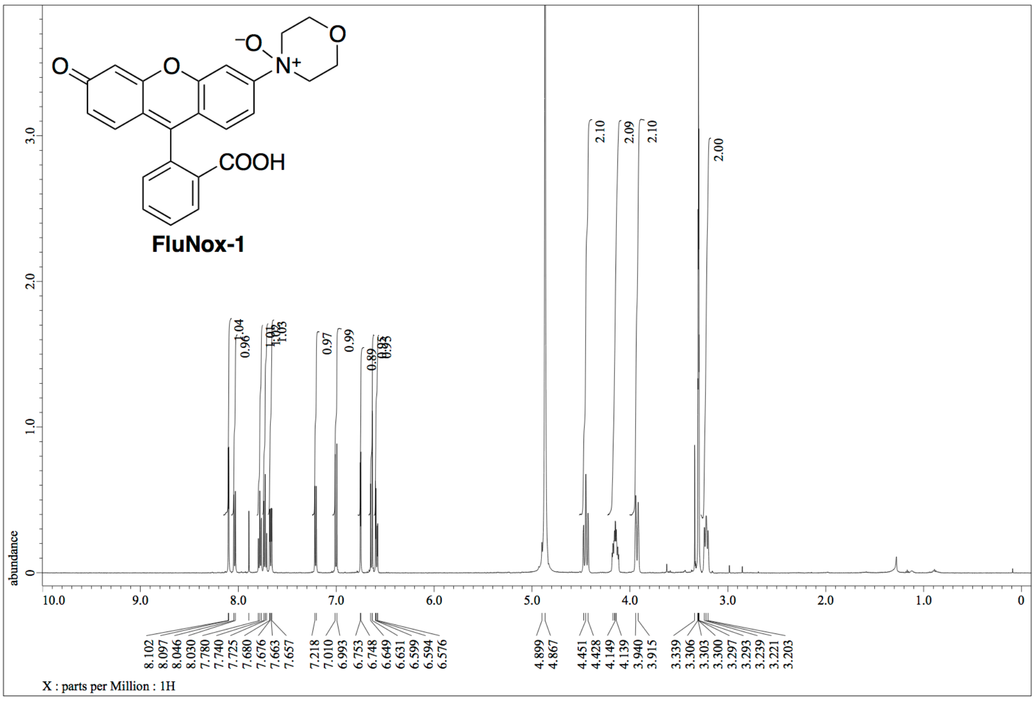
FluNox-1
COOH
X : parts per Million : 1H
abundance

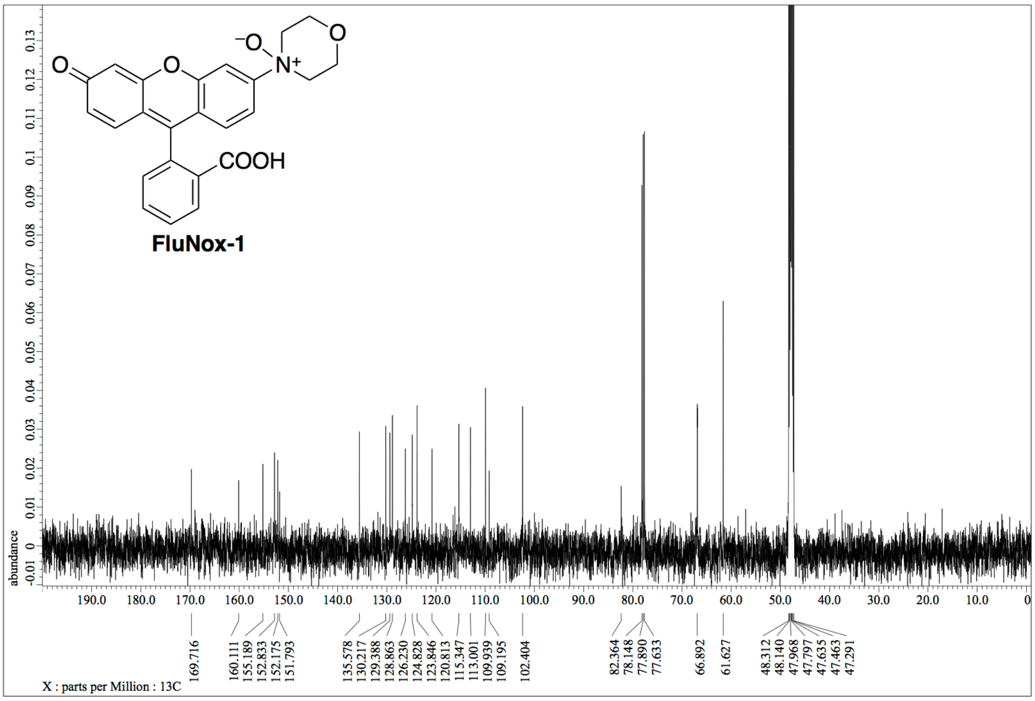
FluNox-1
COOH
X : parts per Million : 13C
abundance

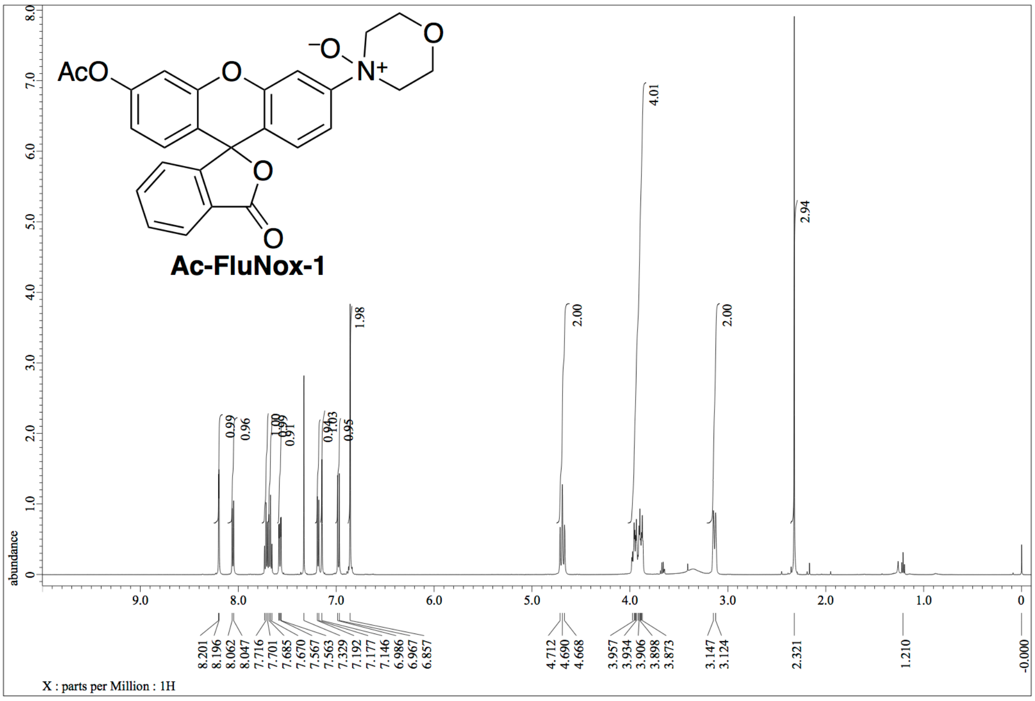
AcO
Ac-FluNox-1
abundance
X : parts per Million : 1H
8.201
8.196
8.062
8.047
7.716
7.701
7.685
7.670
7.567
7.563
7.329
7.192
7.177
7.146
6.986
6.967
6.857
4.712
4.690
4.668
3.957
3.934
3.906
3.898
3.873
3.147
3.124
2.321
1.210
-0.000
0.99
0.96
1.98
2.00
4.01
2.00
2.94

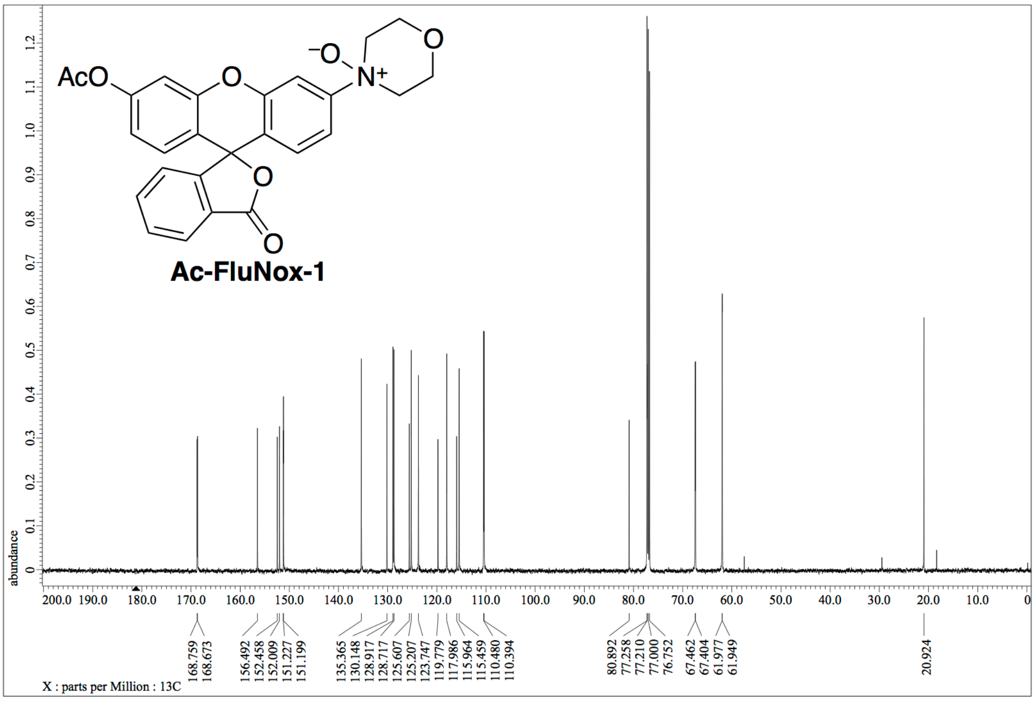
AcO
Ac-FluNox-1
abundance
X : parts per Million : 13C
168.759
168.673
156.492
152.458
152.009
151.227
151.199
135.365
130.148
128.917
128.717
125.607
125.207
123.747
119.779
117.986
115.964
115.459
110.480
110.394
80.892
77.258
77.210
77.000
76.752
67.462
67.404
61.977
61.949
20.924

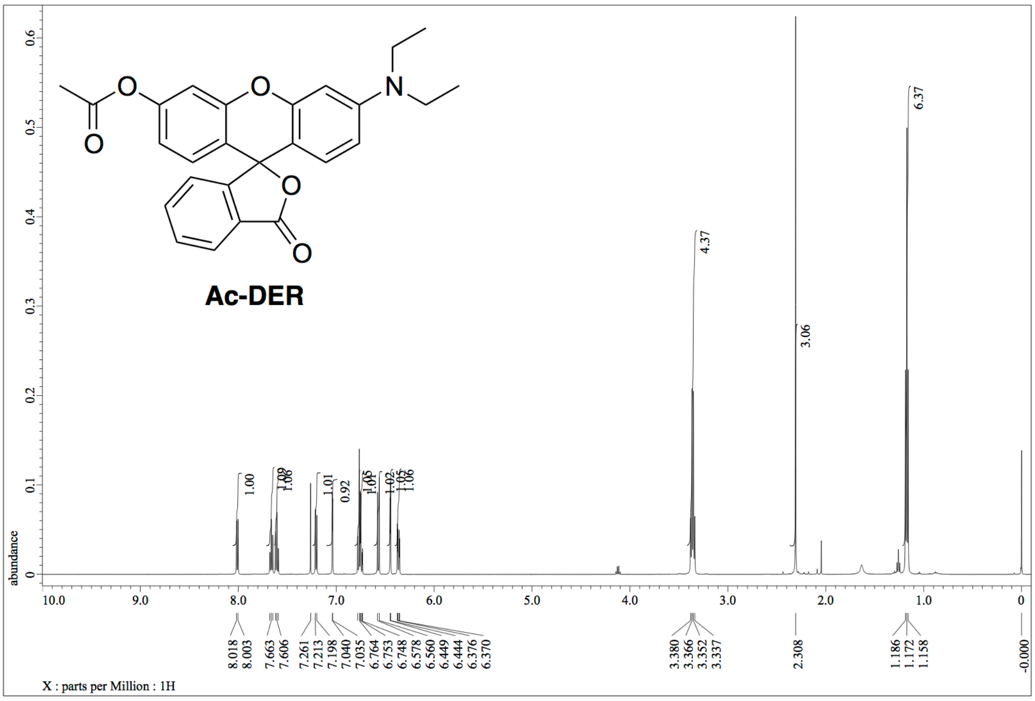
Ac-DER
X : parts per Million : 1H
abundance

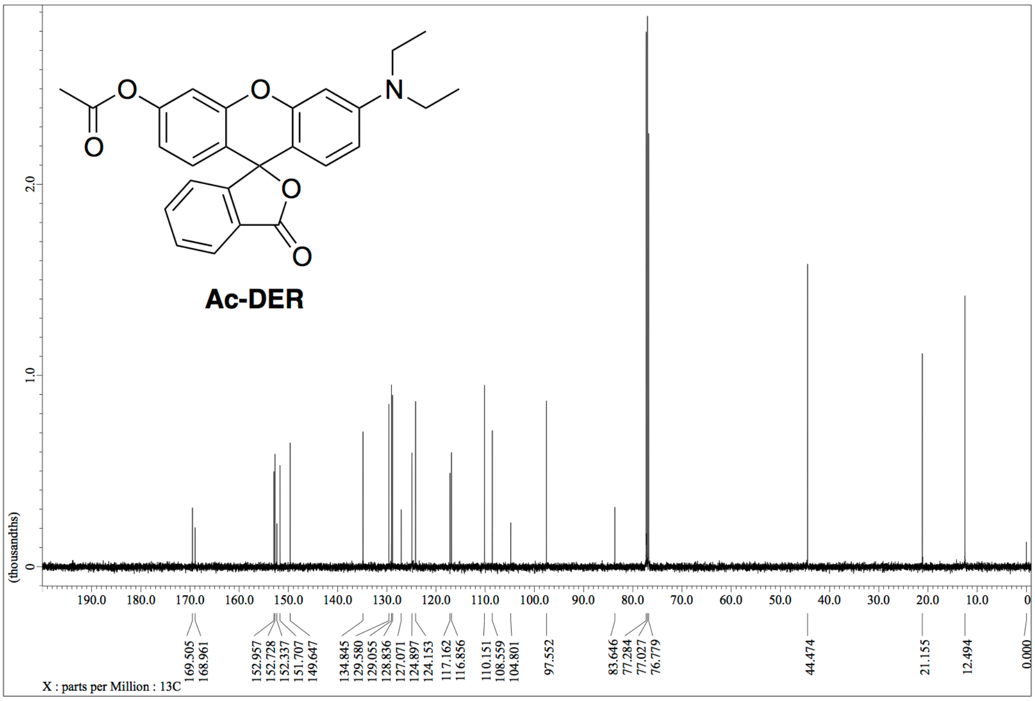
Ac-DER
X : parts per Million : 13C
(thousandths)

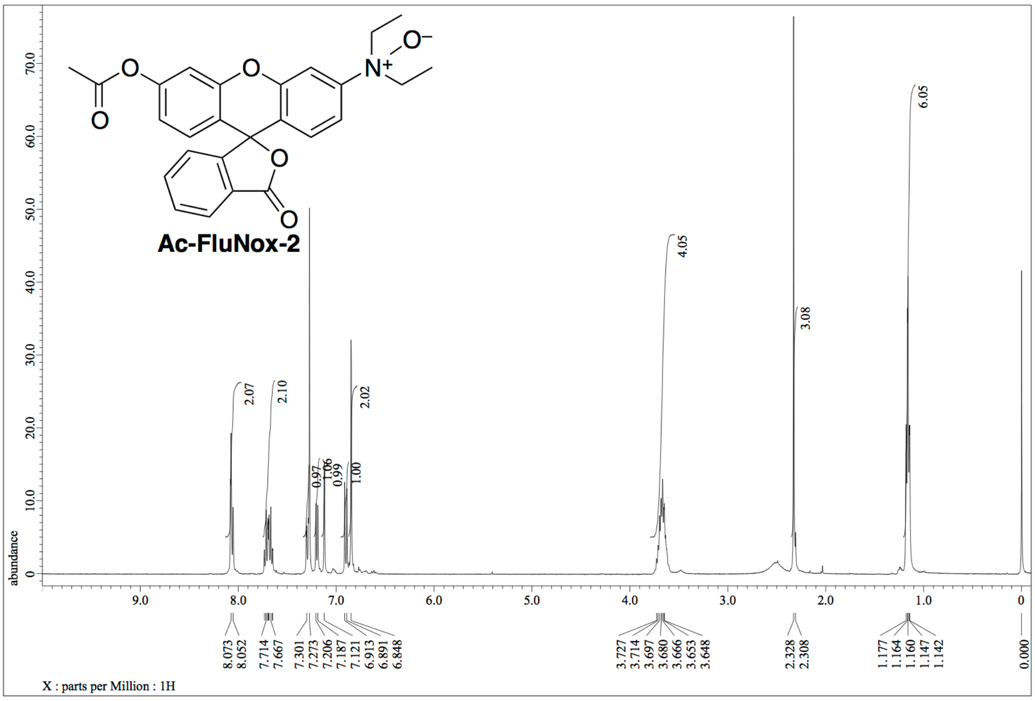
Ac-FluNox-2
abundance
X : parts per Million : 1H
8.073
8.052
7.714
7.667
7.301
7.273
7.206
7.187
7.121
6.913
6.891
6.848
3.727
3.714
3.697
3.680
3.666
3.653
3.648
2.328
2.308
1.177
1.164
1.160
1.147
1.142
0.000
2.07
2.10
0.97
1.06
0.99
1.00
2.02
4.05
3.08
6.05

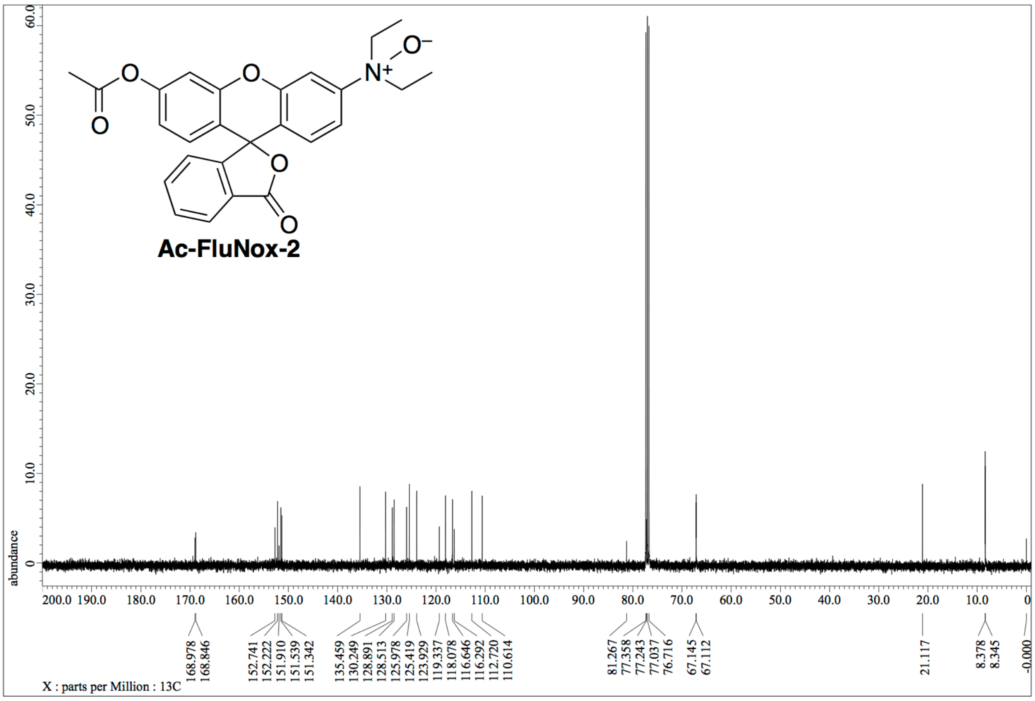
Ac-FluNox-2
abundance
X : parts per Million : 13C
168.978
168.846
152.741
152.222
151.910
151.539
151.342
135.459
130.249
128.891
128.513
125.978
125.419
123.929
119.337
118.078
116.646
116.292
112.720
110.614
81.267
77.358
77.243
77.037
76.716
67.145
67.112
21.117
8.378
8.345
-0.000

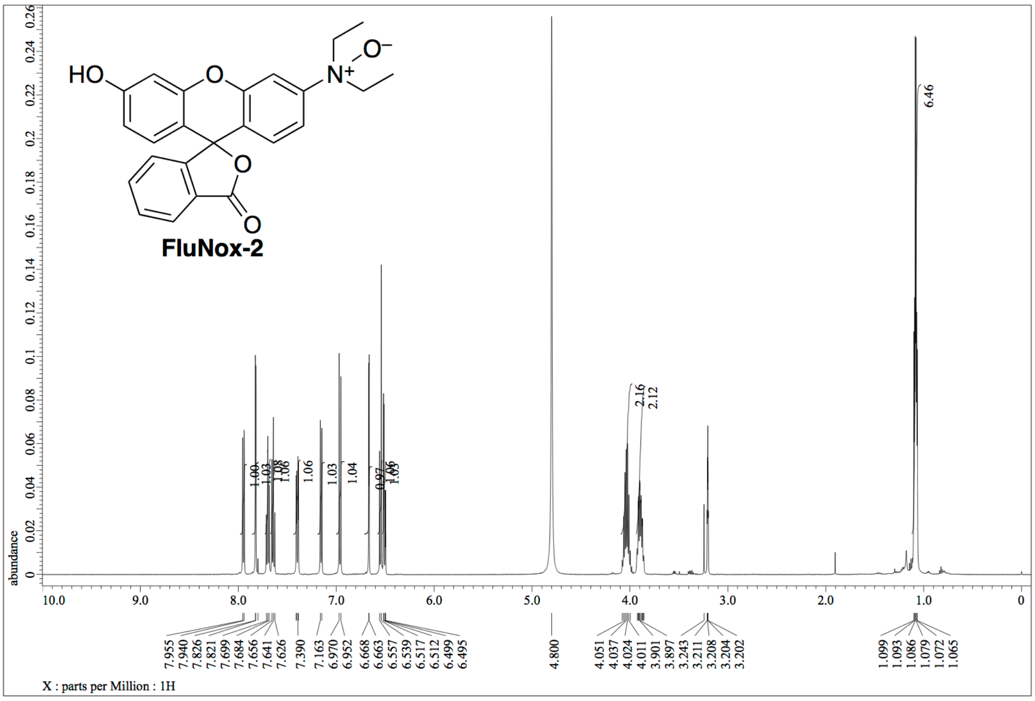

HO
O
N+
O−
O
O
FluNox-2
abundance
X : parts per Million : 1H

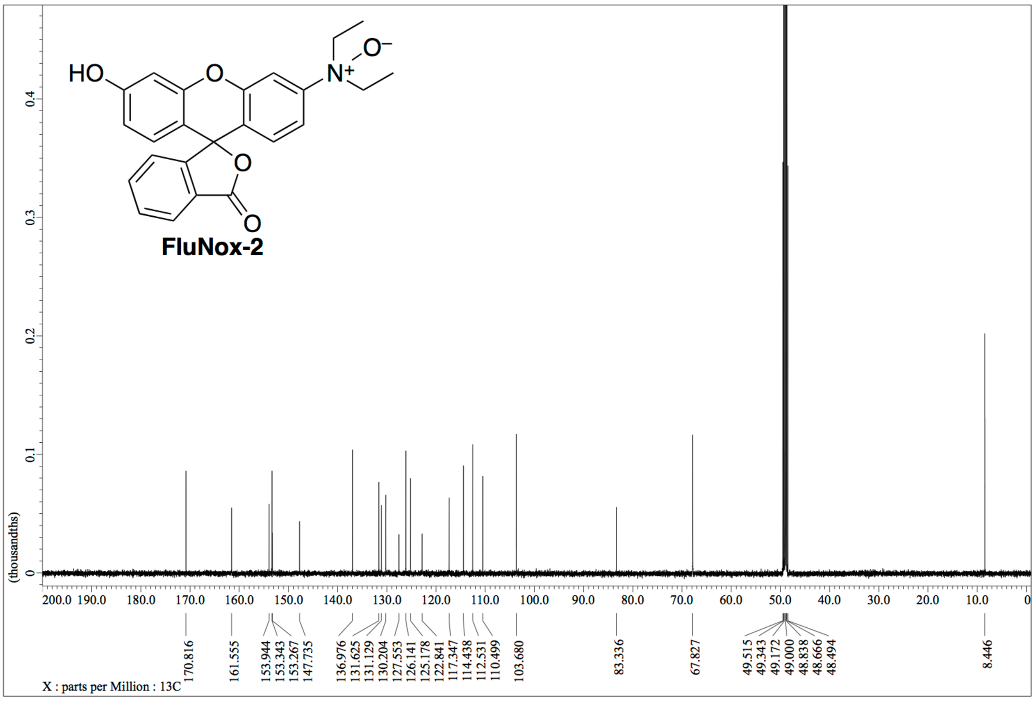

HO
O
N+
O−
O
O
FluNox-2
(thousandths)
X : parts per Million : 13C

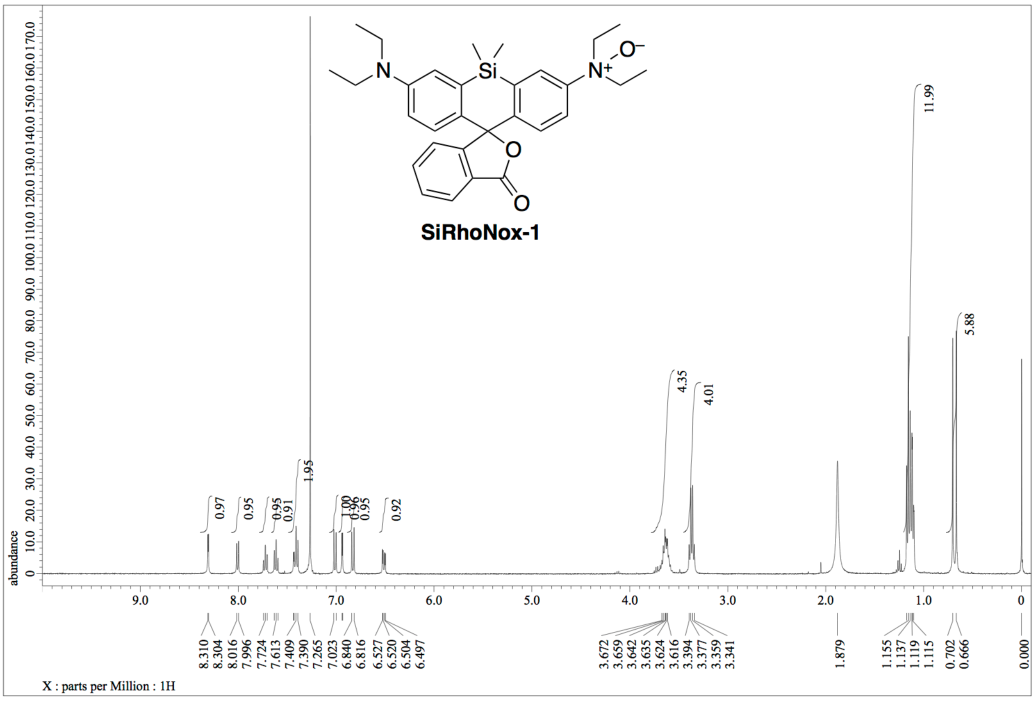

SiRhoNox-1
abundance
X : parts per Million : 1H
0.97
0.95
0.95
0.91
1.95
1.00
0.96
0.95
0.92
4.35
4.01
11.99
5.88
8.310
8.304
8.016
7.996
7.724
7.613
7.409
7.390
7.265
7.023
6.840
6.816
6.527
6.520
6.504
6.497
3.672
3.659
3.642
3.635
3.624
3.616
3.394
3.377
3.359
3.341
1.879
1.155
1.137
1.119
1.115
0.702
0.666
0.000

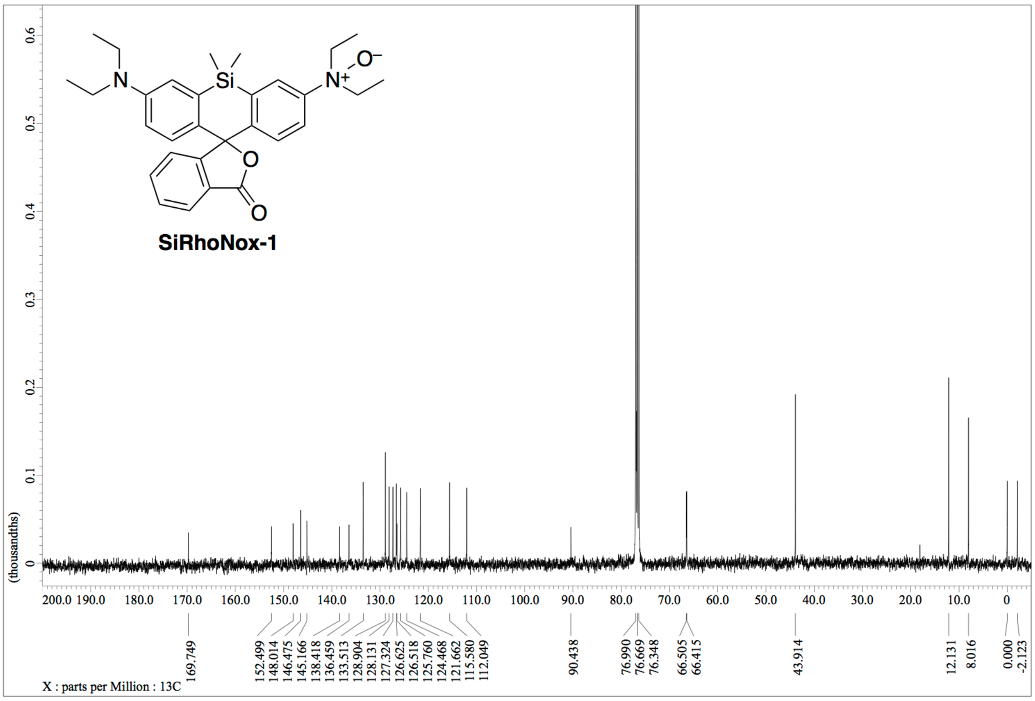

SiRhoNox-1
(thousandths)
X : parts per Million : 13C
169.749
152.499
148.014
146.475
145.166
138.418
136.459
133.513
128.904
128.131
127.324
126.625
126.518
125.760
124.468
121.662
115.580
112.049
90.438
76.990
76.669
76.348
66.505
66.415
43.914
12.131
8.016
0.000
-2.123
